# Ecosystem services potential is declining across European capital metropolitan areas

**DOI:** 10.1038/s41598-024-59333-8

**Published:** 2024-04-17

**Authors:** Artan Hysa, Roland Löwe, Juergen Geist

**Affiliations:** 1 Aquatic Systems Biology, School of Life Sciences, Technical University of Munich, 85354 Freising, Germany; 2 Department of Environmental and Resource Engineering, Technical University of Denmark, Lyngby, Denmark

**Keywords:** Nature-based solutions, Sustainable development goals, Blue-green infrastructure, Urban development, European studies, Freshwaters, Ecosystem services, Sustainability

## Abstract

Ecosystem services (ES) are essential to sustainable development at multiple spatial scales. Monitoring ES potential (ESP) at the metropolitan level is imperative to sustainable cities. We developed a procedure for long-term monitoring of metropolitan ESP dynamics, utilizing open-source land use land cover (LULC) data and the expert matrix method. We compared the ESP results of 38 European Capital Metropolitan Areas (ECMA) regarding biodiversity integrity, drinking water provision, flood protection, air quality, water purification, and recreation & tourism. Our results show significant declines in ESP across ECMA due to LULC alteration between 2006, 2012, and 2018. We found that ECMA in post-socialist European countries like Poland (Warszawa) have experienced high rates of land use transformation with a remarkable impact on ESP. Surprisingly, we found that Fennoscandinan ECMA, like Helsinki, Stockholm, and Oslo which lead the cumulative ESP ranking, faced the ESP reduction of the highest impact in recent years. The correlation analysis of ESP dynamics to urban expansion and population growth rates suggests that inattentive urbanization processes impact ESP more than population growth. We unveil the implications of our results to the EU and global level agendas like the European Nature Conservation Law and the Sustainable Development Goals.

## Introduction

Ecosystem services (ES) are essential to human well-being and sustainable development at global, regional, and local scales. However, most ecosystem services (ES) assessment studies focus on global, national, or regional levels. Surprisingly, studies targeting the urban scale are underrepresented despite the bold controversy about balancing urbanization growth and the enhanced human well-being needs by society^1^. The underrepresentation of cities among ES studies is surprising when considering that initiatives stressing the urgency for more research on urban ES are not very recent^2^. According to our brief search of available literature indexed in Web of Science, during the last decade (between 1 January 2013 and 31 December 2022), 24,791 articles included “ecosystem services” either in their title, abstract, or keywords. Among them, only 17% used the keywords: “urban”, “city”, or “metropolitan”. This share is more than three times below the global population percentage living in urban areas, as reported by World Bank (WB) statistics, at 56.5%^3^.

According to the WB database, European Union (EU) member states have an average of 75% of the urban population share, led by Belgium, with 98%, and Slovakia recording the lowest share of 54%^3^. The average annual change in urban population share among all European Countries (including the non-EU countries) is almost half (0.23%) of the global average (0.44%). However, the yearly increase in urban population shares in South European countries like Albania (0.91%), Portugal (0.55%), and Türkiye (0.49%) is still above the global average and generates significant spatial pressures within the respective metropolitan areas (see Supplementary data, Table S4).

Urban expansion rates are reported to be higher than the respective population growth, indicating a dominating urban sprawling approach against compacted urban development^4,5^. For instance, the global average annual urban sprawl rates between 1990 and 2014 are almost ten times higher (4%)^6^ than the global average urban population growth rate of the same period^3^. The processes of urban expansion result in land use land cover (LULC) alterations, which directly impact the ES provision capacities in metropolitan zones.

The area between the urban and rural lands, known as the wildland-urban interface (WUI), defines a fragile zone under continuous transformation pressures. At the European scale, where three-quarters of EU citizens live in cities, urban and rural linkages are becoming more assertive, elevating the pressure on natural and semi-natural habitats across the territory^7^. Thus, assessing ES potential within the metropolitan regions is imperative to prioritize land allocation for new urban development and safeguarding critical landscapes vital for a sustainable and resilient metropolitan life. This becomes even more critical while considering the emerging metropolitan challenges under the pressures of climate change and the indispensable regulating capacities of ES.

ES can compensate for several social-ecological public health drawbacks caused by urbanization processes^8^. The impact of ES by natural urban areas on the physical and mental health of inhabitants is vital^9^, as evidenced in significant social disturbances during the COVID-19 pandemic^10^. Urban green spaces are reported as proper areas for physical activities and healthier social interactions among people, including disadvantaged groups^11,12^. Furthermore, ES can mitigate the impact of climate change on metropolitan regions like urban heat island formation, flooding, heat stress, and the related microclimate^13^. Ecosystem-based adaptation approaches use ES and biodiverse environments to compensate for the climate change impacts at the local level^14^. A suitable urban environment for functional ES can combat metropolitan regions' ecological footprint, contributing to more resilient, healthier, and citizen-friendly cities. At the same time, reducing urban ES qualities also involves long-term financial costs and can directly affect many other social and cultural systems^15^.

In developed metropolitan areas, including the EU member states, starting from 2013, there has been a considerable increase in the consciousness of ES’s importance in planning nature-based solutions (NBS) for sustainable metropolitan regions^16^. However, implementing ES and NBS principles is not an easy task, facing many socio-political challenges^17,18^. Nevertheless, securing the continuity of the ES provision, in the long run, is imperative, bearing in mind that Europe has been a hotspot of urban sprawl and land transformation trends during the last three decades^6^. Regions that include metropolitan territories of a broad development stages gradient and diverse socio-political backgrounds, like the European continent, are of great interest in enabling sensitive comparisons between the metropolitan development tendencies and the ES potential dynamics.

Referring to the available literature, only a few studies compare ES provision capacities of specific metropolitan areas^19^. For example, cities like Barcelona, Berlin, Helsinki, Salzburg, and Stockholm have been comparatively analyzed in two different studies^20,21^. The earlier study relied on Corine Land Cover (CLC) data^21^, which has a low resolution for urban scale analysis, while the latter utilized Urban Atlas (UA) data, which provides geospatial information at a finer scale^20^. The only large-scale analysis comparing the ES capacities of large metropolitan areas within EU-27 member states dates back to a decade ago^22^. All of the above studies reported static analysis based on the geospatial data of 2006 (UA), without any temporal analysis on the ES provision change relying on more than one period’s records. Furthermore, it only included cases from EU member states, not considering the ES provision dynamics in developing metropolitan areas in the remaining European countries (non-EU). To our knowledge, no previous study compares the spatiotemporal dynamics of ES potential across European metropolitan areas using fine-scale geospatial data at a pan-European level.

In this context, our study is the first to quantify and compare the ES potential (ESP) of 38 European capital metropolitan areas (ECMA) of the EU and non-EU member states. The current availability of UA data for three consecutive periods (2006, 2012, 2018) enables a spatiotemporal comparison among ESP of selected cases for the first time. Furthermore, unlike previous studies, we perform the ESP analysis on two levels: the urban and the metropolitan scale. On the other hand, we provide a revised version of the Expert Matrix method, which was initially developed as an ESP assessment methodology utilizing CLC data at coarse landscape scale^23^. Here, we aim to downscale its spatial scope from coarse landscape to urban scale by shifting from CLC to UA data, including the urban core area.

Specifically, we hypothesize that there has been a significant reduction in the ES potential of metropolitan lands across ECMA during the analyzed periods, mainly due to the new urban development. Furthermore, we predict that ESP dynamics behave differently at the urban (urban core) and metropolitan scales (suburban, WUI, and hinterland). We also expect that the correlation analysis between ESP dynamics and socio-economic variables like urban expansion trends, population growth, total population, population density, metropolitan gross domestic product (GDP), and GDP per capita reveals some common trends and potential differences among ECMA. Our initial assumption is that urban expansion and population growth strongly correlate with ESP reduction. The results will guide our discussion, focusing primarily on the city's socio-political background and underlying policy-making approaches. Relying on our results, we discuss the implications of the ESP changing dynamics to emerging metropolitan challenges at local and global levels.

## Materials and methods

### Study area

**Figure 1:**
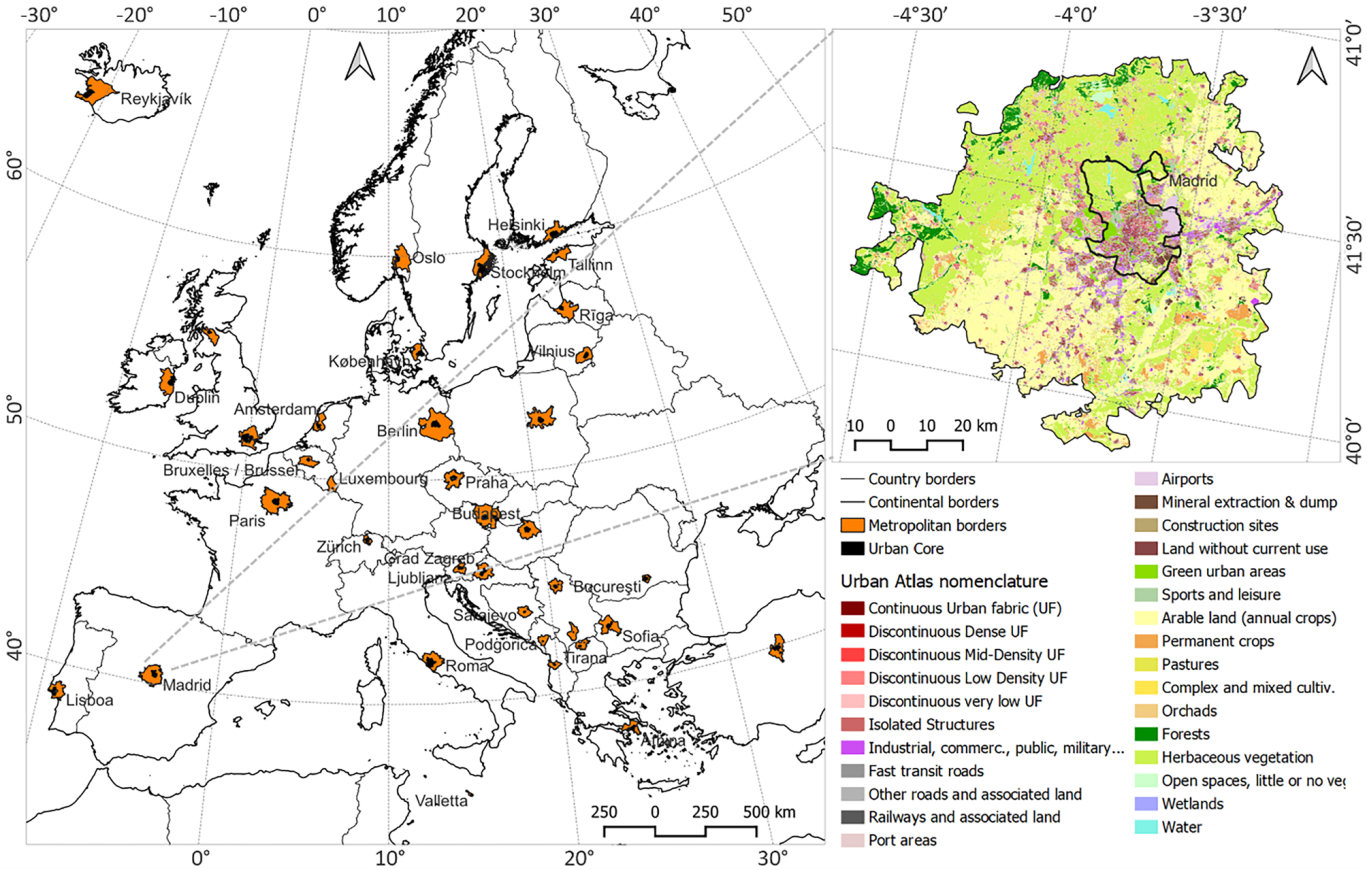
Selected European capital metropolitan areas (ECMA) and Madrid’s UA data for the year 2018, showing both the metropolitan and the urban core boundaries. The maps are produced by the first author using open access administrative map of Europe (retrieved from European Commission via https://ec.europa.eu/eurostat/web/gisco/geodata/reference-data/administrative-units-statistical-units) and the Urban Atlas data (Copernicus land monitoring service via https://land.copernicus.eu/en/products/urban-atlas). The maps are generated via open source software QGIS Desktop 3.24.0.

We focused on the metropolitan areas surrounding the European (EU and non-EU) capital cities. Relying on UA, we first derived the boundary of each study area at the urban and metropolitan levels. Besides the urban core geometry, UA defines a metropolitan border based on the functional urban area (FUA). An example of this pair of boundaries is given in Fig. 1, showing the selected ECMA and a closer view of Madrid, including the UA data and the respective urban and metropolitan boundaries.

In a first step, we analyzed the current ESP in the selected areas. We compared all 38 ECMA, except the capital cities of Belarus, Moldova, and Ukraine, as the respective UA data were not available. In the second phase of the ESP dynamics assessment (between 2012 and 2018), we analyzed the ESP changing trends at all 38 ECMA. In the third phase, when we examined the long-term ESP dynamics between 2006, 2012, and 2018, we had to exclude the capital metropolitan areas of Albania, Bosnia-Herzegovina, Croatia, Iceland, Northern Macedonia, Norway, Montenegro, Kosovo, Serbia, and Switzerland, as the respective UA data of 2006 were not available. Furthermore, during the third phase, we detected no LULC change (2006–2012) information for the metropolitan area of Riga (Latvia), which was therefore also excluded. The remaining 27 ECMA were further processed at the final phase of the study.

### Workflow and the expert-based matrix as a weighting technique for ESP assessment

We were searching for a practical and reasonable method to convert the LULC into ES potential. We found that the expert matrix (EM) method is a relative weighting procedure among ecosystem types (ET) represented by different landscape characterization maps based on normalized experts’ opinions. Unlike economic value-based ES assessment methods, EM remains more sensitive to considering intangible ecosystem values^24^. Yet, setting the weights based on expert preferences needs to be carefully generated following specific procedures to minimize potential bias^25^. Through EM, we could quantify the ES potential (ESP) based on the physical properties of the LULC. Furthermore, considering the diverse economic background among ECMA, we quantified the spatio-temporal dynamics of the ESP change instead of the financial properties. On the other hand, the relative assessment approach brings all cases on a comparable common ground based on the universal qualitative values of ESP.

**Figure 2:**
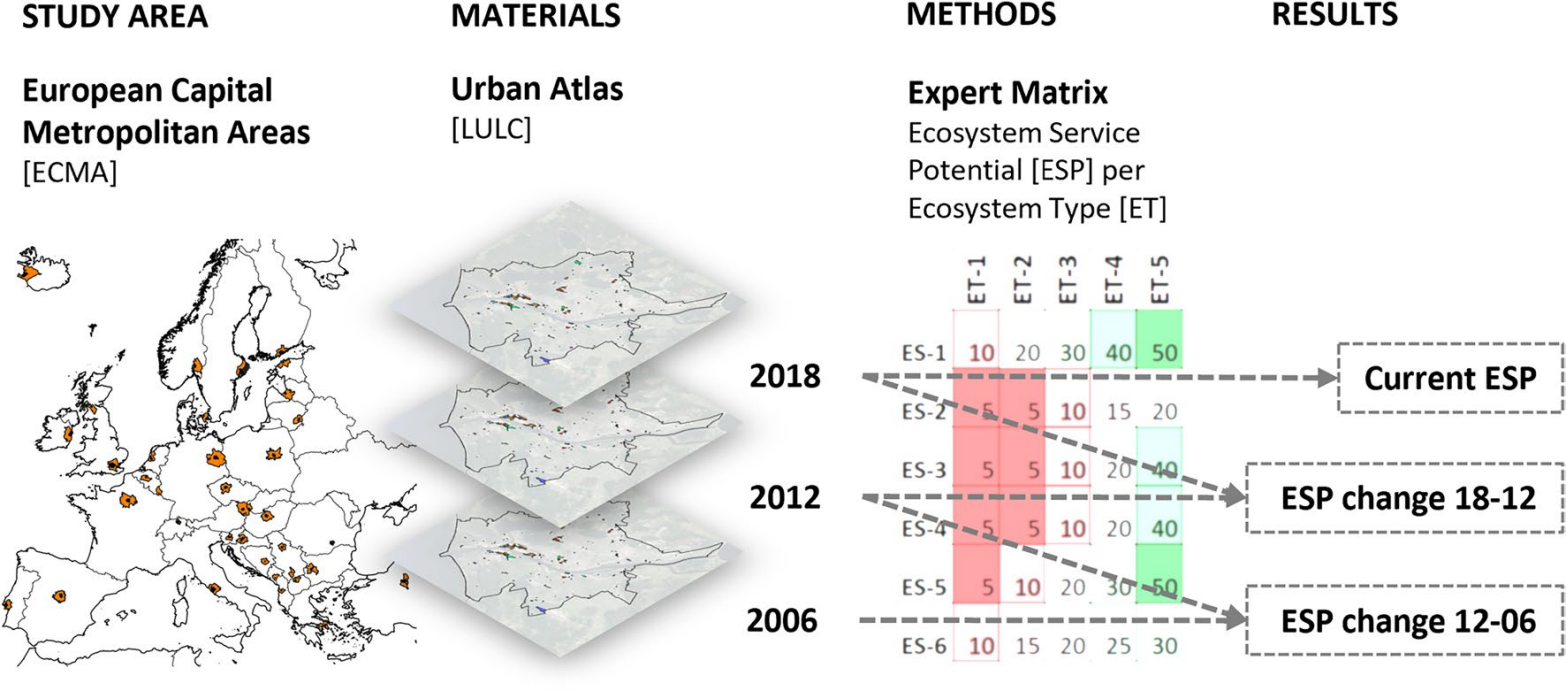
Workflow of the study, including the study area, materials, methods, and results. The maps are produced by the first author using open access administrative map of Europe (retrieved from European Commission via https://ec.europa.eu/eurostat/web/gisco/geodata/reference-data/administrative-units-statistical-units) and the Urban Atlas data (Copernicus land monitoring service via https://land.copernicus.eu/en/products/urban-atlas). The maps are generated via open source software QGIS Desktop 3.24.0.

We utilized the EM method to compare the ecosystem types relative to their significance to different types of ES provision. The EM method is advocated to be a rapid, efficient, flexible, and accessible tool widely used in many studies^25–27^. It was initially developed by Burkhard et al.^28^. Since then, the model has faced different refinements to improve its reliability^27,29^. In our analysis, we utilized a recent version of the matrix developed by Müller et al.^23^, which was generated via a series of workshop sessions among invited experts in the field. The method consists of a scoring procedure that assigns a weighted relative score to each ES and ecosystem type combination based on the potential of the respective ecosystem type to provide the specific ES. It relies on a scoring gradient between 5 (min) and 100 (max). The EM is a critical step in the workflow of our study (see Fig. 2). Yet, we acknowledge the limitations of this method related to potential subjectivity, which in this study is minimized by relying on a validated previous study^23^.

#### Downscaling the expert matrix method

We adapted the original expert matrix developed by Muller et al.^23^, according to the scope and objective of our study. The original matrix identifies 37 ES grouped under four categories: ecosystem integrity (I), provisioning services (P), regulating services (R), and cultural services (C), as specified in the supplementary data. While all initial ES types have close implications with coastal and regional scales, only some are relevant to the urban and metropolitan scales. Thus, we shortlisted six as the most pertinent ES types for the urban context, keeping at least one representative ES from each category.

First, we excluded the ES directly relevant to the coastal and ocean context, like *fish and seafood*, *beach wrack*, and *flotsam*. Then, we excluded ES that are more relevant to the rural areas than the urban and metropolitan areas, like *exergy capture*, *provision of timber*, and *wood fuel*. Finally, we excluded all ES related to agricultural production, like *crops*, *livestock*, and *pollination*. We want to highlight that we do not underestimate the importance of the excluded ES to the broader areas in the metropolitan hinterlands. Nevertheless, here we aimed to focus only on the core ES that have the highest relevancy to the urban context, such as *integrity of biodiversity* (I1), *drinking water* (P1), *flood protection* (R1), *air quality* (R2), *water purification* (R3), and *recreation and tourism* (C1). Our supplementary material provides further details about the rationale of the shortlisted ES (Supplementary Dataset 1).

In the second step, we adapted the ESP values of CLC to the UA nomenclature. First, we used the same values for 15 LULC classes that are equivalent between CLC and UA data. Nevertheless, there are classes in CLC nomenclature that are split into subclasses in UA data and vice versa. For example, CLC nomenclature includes only two classes for urban fabric (continuous and discontinuous). In contrast, UA data splits the discontinuous urban fabric into four subclasses according to settlement density (Supplementary Dataset 1). In such cases, we hierarchically distributed the ESP values among four UA discontinuous urban fabric classes by keeping the overall group average equal to the original ESP value (discontinuous urban fabric). The upper and lower bounds of the hierarchically distributed range are defined by other artificial (continuous urban fabric) and natural (forests, urban greenery, etc.) land cover classes of UA data to reduce bias.

On the other hand, CLC nomenclature consists of five classes of wetlands and five classes of water surfaces, while the UA data consists of only one class of wetland and one class of water surfaces. In this case, we first disregarded water surfaces irrelevant to the urban context (ex., Open coastal waters). Then, we used the average ESP values of the remaining surface waters (ex., Inland waters, and inner coastal waters in the case of coastal metropolitan areas). We provide as supplementary data further detailed information on how we have derived the final list of ecosystem types and their respective ESP for each ES (Supplementary information file). As a result, we derived the final ESP scoring we used in our analysis, as presented in Fig. 4.

#### The updated expert matrix

**Figure 3:**
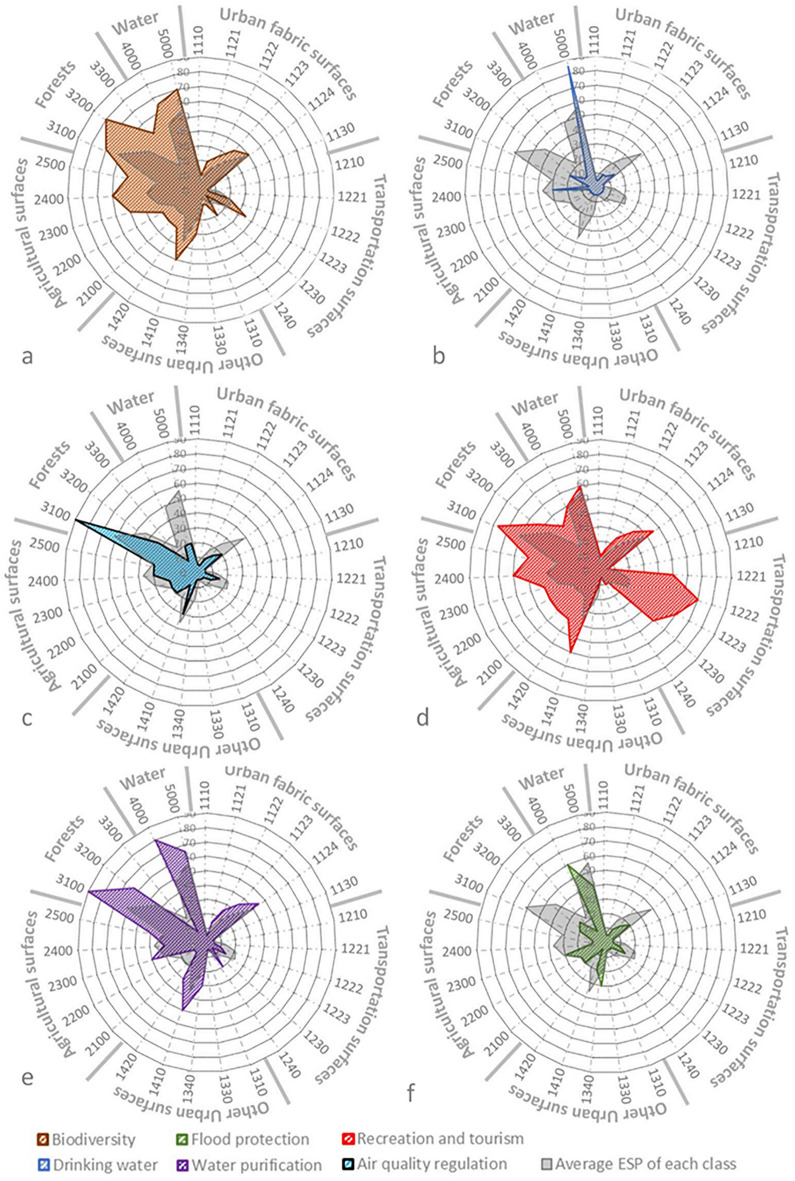
ESP values of each ecosystem type for the selected ES: (**a**) biodiversity integrity, (**b**) drinking water, (**c**) water purification, (**d**) air quality, (**e**) recreation and tourism, and (**f**) flood protection overlapped to average ESP values shown In grey. Ecosystem type relies on the UA nomenclature as follows: 1110: Continuousu urban fabric (SL > 80%), 1121: Discontinuous dense urban fabric, 1122: Discontinuous medium density urban fabric, 1123: Discontinuous low-density urban fabric, 1124: Discontinuous very low-density urban fabric, 1130: Isolated structures, 1210: Industrial, commercial, public, military and private, 1221: Fast transit roads and associated land, 1222: Other roads and associated land, 1223: Railways and associated land, 1230: Port areas, 1240: Airports, 1310: Mineral extraction and dump sites, 1330: Construction sites, 1340: Land without current use, 1410: Green urban areas, 1420: Sports and leisure facilities, 2100: Arable land (annual crops), 2200: Permanent crops, 2300: Pastures, 2400: Complex and mixed cultivation patterns, 2500: Orchards, 3100: Forests, 3200: Herbaceous vegetation associations, 3300: Open spaces with little or no vegetations, 4000: Wetlands, 5000: Water.

Following the abovementioned rationale, we derived the updated matrix adapted to our study's scope and objective. The radar charts in Fig. 3 rely on the detailed Table S2 (see Supplementary Information), where the color gradient of green-white-red helps to visually differentiate between low and high ESP significance of ES and ecosystem type pairing. For instance, fast transit roads (1221) and port areas (1230) have the lowest potential to provide ES of biodiversity integrity (I1) (see Fig. 3a). Meanwhile, forests (3100), wetlands (4000), and water surfaces (5000) hold the highest ecosystem services potential of all ES types.

Referring to the average ESP values of each ecosystem type (the grey area in the radar charts of Fig. 3), industrial, commercial, public, military, and private buildings (1210), isolated structures (1130), construction sites (1330), mineral extraction and dump sites (1310), and continuous urban fabric (1110) are among the ecosystem types that have the lowest capacities to provide ES. Forests (3100), wetlands (4000), water surfaces (5000), and herbaceous vegetation (3300) are the ecosystem types that have the highest potential for the shortlisted ES at the metropolitan level.

It is worth highlighting that among artificial surfaces, only the transportation network has an above-average ESP regarding recreation and tourism (see Fig. 3d). Overall, the ESP regarding recreation and tourism (Fig. 3d) and biodiversity integrity (Fig. 3a) are above the average ESP of all six ES types. Regulating services like air quality (Fig. 3c) and water purification (Fig. 3e) are close to the average ESP. In contrast, ESP values by all ecosystem types regarding drinking water (Fig. 3b) and flood protection (Fig. 3f) remain remarkably below the average ESP values.

### UA data as information on ecosystem types

Land use and land cover maps are the most frequently used mapping units in recent ES assessment studies^30^. LULC change significantly impacts the Ecosystem's capacity to provide services^31^. Comparison between previous and current LULC states is widely used in literature to reveal the artificialization processes at peri-urban and metropolitan levels^32^. Thus, long-term LULC monitoring data can be utilized to understand ES potential dynamics. Previous studies have utilized open-source LULC data like Corine Land Cover (CLC) to quantify the ES potential at the continental scale^23,33^. However, CLC data is too coarse in scale to inform studies at urban and metropolitan scales. Nevertheless, besides the CLC data, the European Commission provides UA data for the European metropolitan areas at a finer spatial scale.

UA is an open-source LULC data provided by Copernicus Land Monitoring Service by the European Environment Agency. It is divided into patches with homogeneous land use, classified according to the nomenclature of 17 urban and 10 rural LULC classes with a minimum mapping unit of 0.25 ha and 1 ha, respectively. Even though UA data has certain limitations regarding resolution at the fine scale, it has a thematic accuracy above 80%^34^. Thus, it is particularly useful for pan-European metropolitan-level investigations like our study. Furthermore, UA is updated every six years, delivering up-to-date information on LULC, currently covering 2006, 2012, and 2018^35^.

Previous studies have utilised UA data as reliable geospatial information to analyse the landscape structure at metropolitan and urban levels focusing on diverse environmental challenges^36,37^, including ecosystem services provision^22^. Furthermore, it delivers a second product on LULC change based on the differences between two consecutive periods. Currently, two LULC change packages are available, comparing the 2012–2018 and 2006–2012 periods. In our study, we first used the UA data 2018 to compare all ECMA by their current ESP. While the UA change products enabled us to reveal the ESP dynamics across three different periods. Consequently, we could differentiate between metropolitan areas that have faced high rates of ESP reduction and ECMA that have slightly improved.

### Indicators of dynamics of ecosystem service potential (ESP)

In the following, we define indicators for ESP dynamics. These will be applied to ESP for individual ecosystem services and the averaged ESP, as illustrated in Fig. 3.

#### Cumulative ES potential at metropolitan and urban level

We used ESP_cum_ as a cumulative indicator to compare ECMA regarding their ES potential [Eq. (1)] based on the current LULC data (UA, 2018). The analysis is performed at the urban (urban core boundary) and metropolitan level (FUA boundary).

1 $$ES{P}_{cum}=\sum_{p=1}^{n}\frac{(ES{P}_{p}\times {A}_{p})}{{A}_{tot}}$$

where *ESP*_*cum*_ is the cumulative ES potential including all patches within the study area (see Table S2), ESPp is the ES potential of patch *p* based on the current UA map, *A*_*p*_ is the area of patch *p*, *A*_*tot*_ is the total surface of the study area, and *n* is the total number of patches within the study area.

#### ESP dynamics at patch level (2012–2018)

We started the ESP dynamics (ESPD) assessment at the patch level, comparing the old LULC type of 2012 and 2018 [Eq. (2)]. UA change products include only the land patches transformed during the respective six years (see Fig. S1). Its attribute table consists of the altered patch's previous and recent LULC type. At the same time, this step delivers the number of changing patches as an indicator of the number of attempts of LULC alteration at each ECMA, translated into ESP change. We decide to present the patch-level *ESPD*_*p*_ via a joint plotting technique integrating both box plotting and strip-chart (see Fig. 6).

2 $$ESP{D}_{p}=(ES{P}_{new.p}-ES{P}_{old.p})$$

where *ESPD*_*p*_ is ecosystem service potential dynamics at the patch level, *ESP*_*new.p*_ is the current ecosystem service potential of the changed patch and *ESP*_*old.p*_ is the previous ecosystem service potential of the altered patch.

#### Cumulative, mean, and impact of ESP dynamics between 2006 and 2018

ESPD calculation enables the measurement of three parameters: (i) the total change of ES potential (*ESPD*_*cum*_), (ii) the average ESP change per patch (*ESPD*_*mean*_), and (iii) the absolute impact of ESP change (*ESPD*_*ch.i*_). *ESPD*_*cum*_ indicates the cumulative change of ESP based on the ratio of the surface area of changed patches to the total study area [Eq. (3)]. *ESPD*_*mean*_ is the average *ESPD*_*p*_ at the patch level, indicating the average effect that land alteration decisions (by each city administration) have had on ESPD [Eq. (4)]. While *ESPD*_*ch.i*_ refers to the absolute impact of LULC change on ESP of the study area measured as a ratio of *ESPDp* multiplied by the changed patch area to the total area of all changed patches [Eq. (5)]. We separately applied the analysis at urban and metropolitan levels to reveal each case's dominant ESP dynamics. Since negative numbers will characterise the ESP reduction, the lower the *ESPD*_*cum*_, *ESPD*_*mean*_*,* and *ESPD*_*ch.i*_ results, the higher the impact of LULC change on ES provision within the study area.

3 $$ESP{D}_{cum}=\sum_{p=1}^{n}ESP{D}_{p}\frac{{A}_{p}}{{A}_{tot}}$$

4 $$ESP{D}_{mean}=\sum_{p=1}^{n}\frac{(ES{PD}_{p})}{n}$$

5 $$ESP{D}_{ch.i}=\sum_{p=1}^{n}ESP{D}_{p}\frac{{A}_{p}}{{A}_{tot.ch}}$$

where *ESPD*_*cum*_ is the ES potential change of all patches within the study area, *ESPD*_*mean*_ is the ratio of the total ESP change per total count of changed patches, *ESPD*_*p*_ is ecosystem service potential dynamics at the patch level, *A*_*tot*_ is the total surface area of the study area, *A*_*tot.ch*_ is the total surface area of changed LULC patches within the study area, *ESPD*_*ch.i*_ is the absolute impact of changed LULC patches, *A*_*p*_ is the area of patch *p, n* is the total number of patches.

#### Calculation of indicators

**Figure 4:**
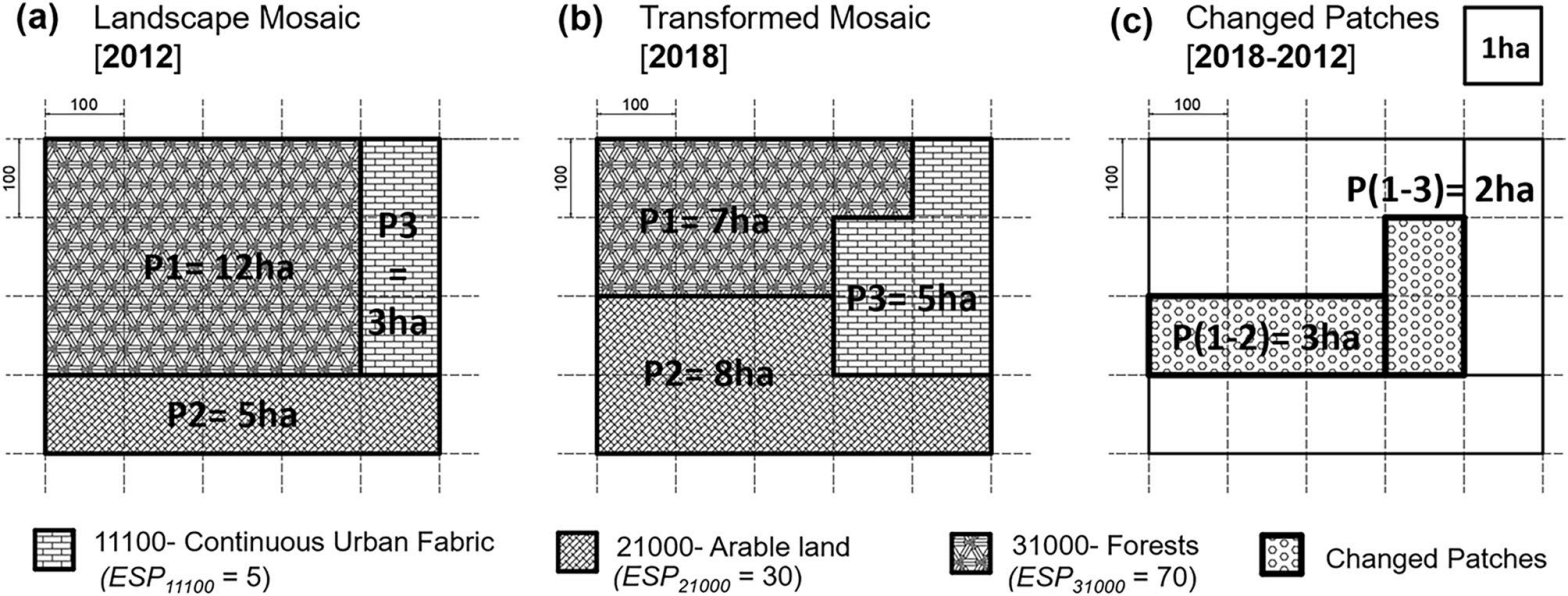
A hypothetical example of a Landscape mosaic composed of three patches (P1—Forests, P2—Arable land, and P3—Continuous urban fabric) in two different years (**a** 2012 and **b** 2018), including the changed patches (P1-2 and P1-3, in **c**). One square unit is 1 ha.

We provide a hypothetical case of a landscape mosaic to demonstrate the calculation of the indicators introduced in the previous sections (Fig. 4), which presents a simplified landscape mosaic configuration comprising three LULC patches (P1, P2, and P3). We randomly assigned a specific LULC type to each patch from UA nomenclature, such as 31,000—Forests (P1), 21,000—Arable land (P2), and 11,100—Continuous urban fabric (P3). Consequently, we used the respective ESP values of the selected ES (I1-Biodiversity integrity, see Fig. 3a) to calculate the indicators. To simplify the information, we selected one square unit as 1 ha. We use a real example of a transformed patch located in the southern suburban area of Tirana (Albania) to exemplify the "patch” notion and how ESPDcum is calculated (see Supplementary Information, Fig. SI). The “patch” is the most frequently used minimum unit extent in ES assessment studies^30^.

Table 1 presents the calculation of each indicator based on the equations introduced earlier. First, we calculated the ecosystem service potential of each patch (ESPp). For example, the first patch (P1) was covered by forests with an area of 12 ha in 2012. The ESP value (matrix value) of 11,100-forests is 70. Thus, the ESPp in 2012 is calculated as 840. However, 5 ha of P1 has been transformed into urban fabric and arable lands in 2018. Therefore, the ESPp value of P1 in 2018 is reduced to 490. Similarly, we calculate ESPp values of P2 and P3, which record a slight increase due to urban and agricultural land expansion towards forested lands. Yet, the cumulative ESP value (ESPcum) of the whole mosaic has experienced a total reduction of -250 (see Table 1a). We derive the same cumulative value of ESP difference (ESPDcum) when focusing on the transformed patches only via a boolean difference operation (see Fig. 4 and Table 1b).

**Table 1:** Calculation of indicators ESPp & ESPcum (a) and ESPDp & ESPDcum (b) for the hypothetical case presented in Fig. 4.

(a) Patch	ESP	Area 2012	ESPp 2012	Area 2018	ESPp 2018	(b) Patch	Area	ESP 2012	ESP 2018	ESPDp
1	70	12	840	7	490	1–2	3	70	30	− 120
2	30	5	150	8	240	1–3	2	70	5	− 130
3	5	3	15	5	25	ESPD_cum_				− 250
Total	ESP_cum_		1005		755					

## Results

**Figure 5:**
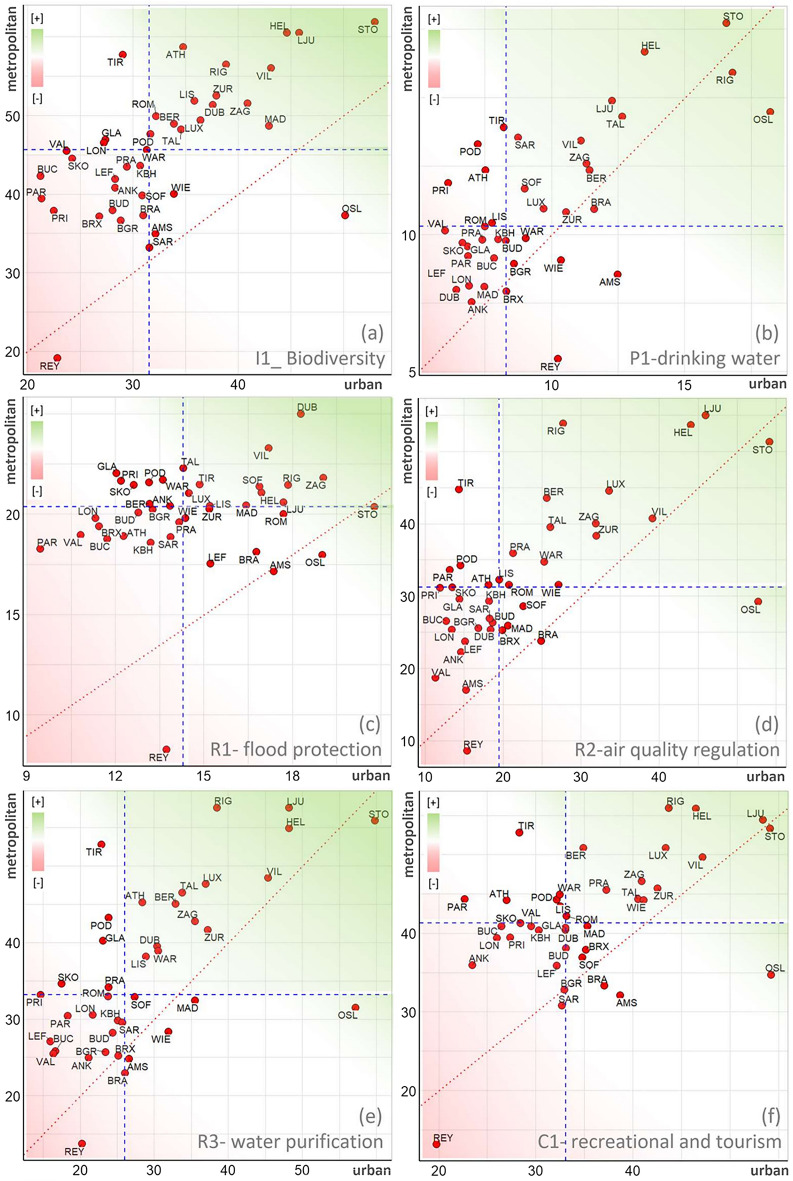
Distribution of ESP_cum_ values of each ECMA at both urban (x-axis) and metropolitan (y-axis) levels for (**a**) I1—biodiversity integrity, (**b**) P1—drinking water availability, (**c**) R1—flood protection, (**d**) R2—air quality regulation, (**e**) R3—water purification, (**f**) C1—recreational and tourism. Red dashed lines indicate equal urban and metropolitan values, while blue dashed lines indicate the respective mean values of each axis. Where: *AMS* Amsterdam, *ANK* Ankara, *ATH* Athina, *BGR* Beograd, *BER* Berlin, *BRA* Bratislava, *BRX* Bruxelles, *BUC* Bucharest, *BUD* Budapest, *DUB* Dublin, *GLA* Glasgow, *HEL* Helsinki, *KBH* København, *LEF* Lefkosia, *LIS* Lisboa, *LJU* Ljubljana, *LON* London, *LUX* Luxembourg, *MAD* Madrid, O*SL* Oslo, *PAR* Paris, *POD* Podgorica, *PRA* Praha, *PRI* Pristina, *REY* Reykjavík, *RIG* Rīga, *ROM* Roma, *SAR* Sarajevo, *SKO* Skopje, *SOF* Sofia, *STO* Stockholm, *TAL* Tallinn, *TIR* Tirana, *VAL* Valletta, *VIL* Vilnius, *WAR* Warszawa, *WIE* Wien, *ZAG* Zagreb, *ZUR* Zürich.

Our findings revealed significant changes in ESP across most ECMA. Between 2012 and 2018 only, ECMA recorded an average LULC change (from natural and semi-natural surfaces to artificial lands) of 78.6 m^2^/ha. Nevertheless, there were specific differences between cases. For example, capital metropolitan areas like Bucharest (Romania) and Ankara (Türkiye) recorded the most significant change, 330 m^2^/ha and 277 m^2^/ha, respectively (see Fig. 10a). In contrast, Reykjavik (Iceland) recorded as low as 8.8 m^2^/ha, which can be considered a positive finding, as Reykjavik had the least *ESPcum* values (see Fig. 5) in our earlier results on the current ES potential (based on UA of 2018).

### Current ESP capacities across ECMA

Most ECMA had higher *ESPcum* at the metropolitan level than the urban (Fig. 5). This is due to the high densities of the urban fabric and other artificial surfaces at the urban level and the vegetated surfaces at the metropolitan level. Nevertheless, Oslo was the only case that recorded higher *ESPcum* values at the urban level than the metropolitan for all six ES. Cities like Zagreb, Riga, and Helsinki were among cities that scored high *ESPcum* values at both urban and metropolitan levels (upper-right corner in scatter plots, Fig. 5) for all ES types. In contrast, cities like London, Ankara, and Reykjavik were constantly within the lowest-performing cases (bottom-left corner). The ES provision potential of Tirana and Podgorica was significantly higher at the metropolitan level than that of the respective urban areas.

For all ES types, Reykjavik recorded the lowest *ESPcum* values at the metropolitan level. This was attributed to the vast abundance of open spaces with little or no vegetation (3300—beaches, dunes, bare rocks, glaciers) at the metropolitan level. Referring to Table S2, the average *ESPcum* of class 3300 is 22 (ranging from 10 to 50), the lowest among surfaces widely found in metropolitan areas. Nevertheless, at the urban level, Reykjavik recorded higher *ESPcum* values regarding drinking water provision (see Fig. 5b).

### Spatio-temporal dynamics of ESP through 12 years period across ECMA

#### ESP dynamics between 2012 and 2018 at patch level

**Figure 6:**
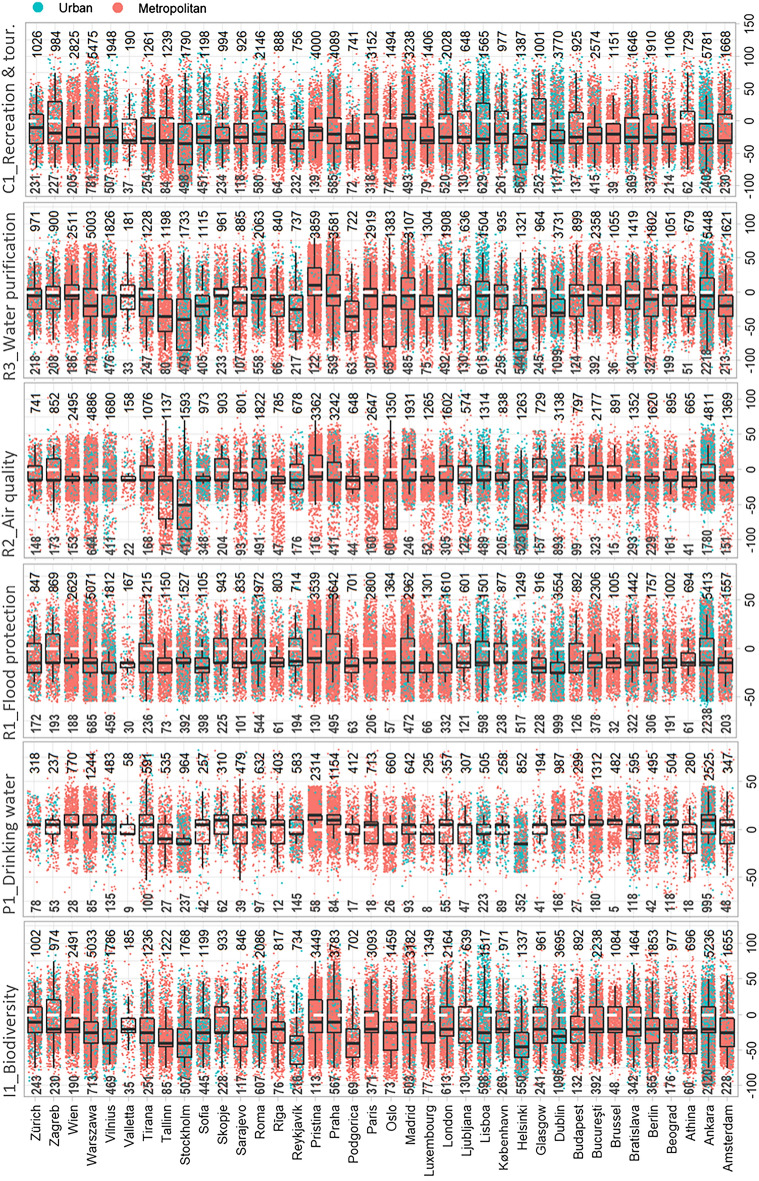
ESPD_p_ of six ES at landscape patch level for all changed surfaces within the selected ECMA (2012–2018). Each point represents the ESPD of a single patch at the urban (blue dots) and metropolitan (red dots) levels, and the count numbers refer to the number of points of non-zero ESPD_p_ value. The threshold line (white dashed) indicates the neutrality state of no change (ESPD = 0). It is a reference line that identifies whether the respective LULC alteration is an improvement or degradation of the respective urban ES. The upper numbers on each chart indicate the total amount of non-zero ESPDp, highlighting the count of only the altered patches that have affected the respected ESPcum, while the lower numbers indicate the changes within the urban core area only.

*Biodiversity integrity* (integrity—I1), *flood protection* (regulating—R1), and *air quality* (regulating—R2) were constantly deteriorating in each ECMA case, as the median *ESPD*_*p*_ values of the respective ES were below the threshold of “no change” (white dashed line, see Fig. 6). While water-related ES, like *drinking water provision* (provisioning—P1) and *water purification* (regulating—R3), were less affected by LULC change as their median values were above or very close to the threshold. It is worth mentioning that P1 was low for all metropolitan areas in absolute terms, as all types of anthropogenic land use affected this ES (see Fig. 5b). On the other hand, R1 was less affected by low-density urban fabric as the permeable surfaces and vegetation layer are more present and significantly contribute to water purification.

ECMA's *recreation and tourism* (cultural—C1) ES provision potential was reduced between 2012 and 2018, dominated by metropolitan areas with low median values, except Madrid, with an above zero median *ESPD*_*p*_ value. Our results show that most LULC changes happened in the metropolitan zone compared to the urban core, which aligns with our initial hypothesis. However, ECMA like Lisbon, Helsinki, Dublin, and Ankara record significantly high LULC changes within the urban core. For instance, 2120 out of 5236 total altered patches in Ankara have been recorded within the urban core (I1-*biodiversity integrity*). Similarly, more than 40% of LULC alteration happened within the urban core of Helsinki, and the remaining occurred within the respective metropolitan zone (Fig. 6).

Most altered patches did not affect *drinking water* ESP dynamics (see Fig. 6). This is complementary to Table S2, as the ESP values of 18 out of 27 LULC classes were assigned the lowest weighted value of “5” in the case of *drinking water* (P1). This leads to many unchanged ESP (zero *ESPD*_*p*_) values despite the change in LULC. It is worth highlighting that cities like Oslo, Stockholm, and Helsinki, the metropolitan areas with the highest ES provision potential (see Fig. 5), were reported among the cases facing the highest degradation during 2012–2018 (see Fig. 6).

#### Cumulative and mean ESPD at metropolitan and urban levels (2012–2018)

**Figure 7:**
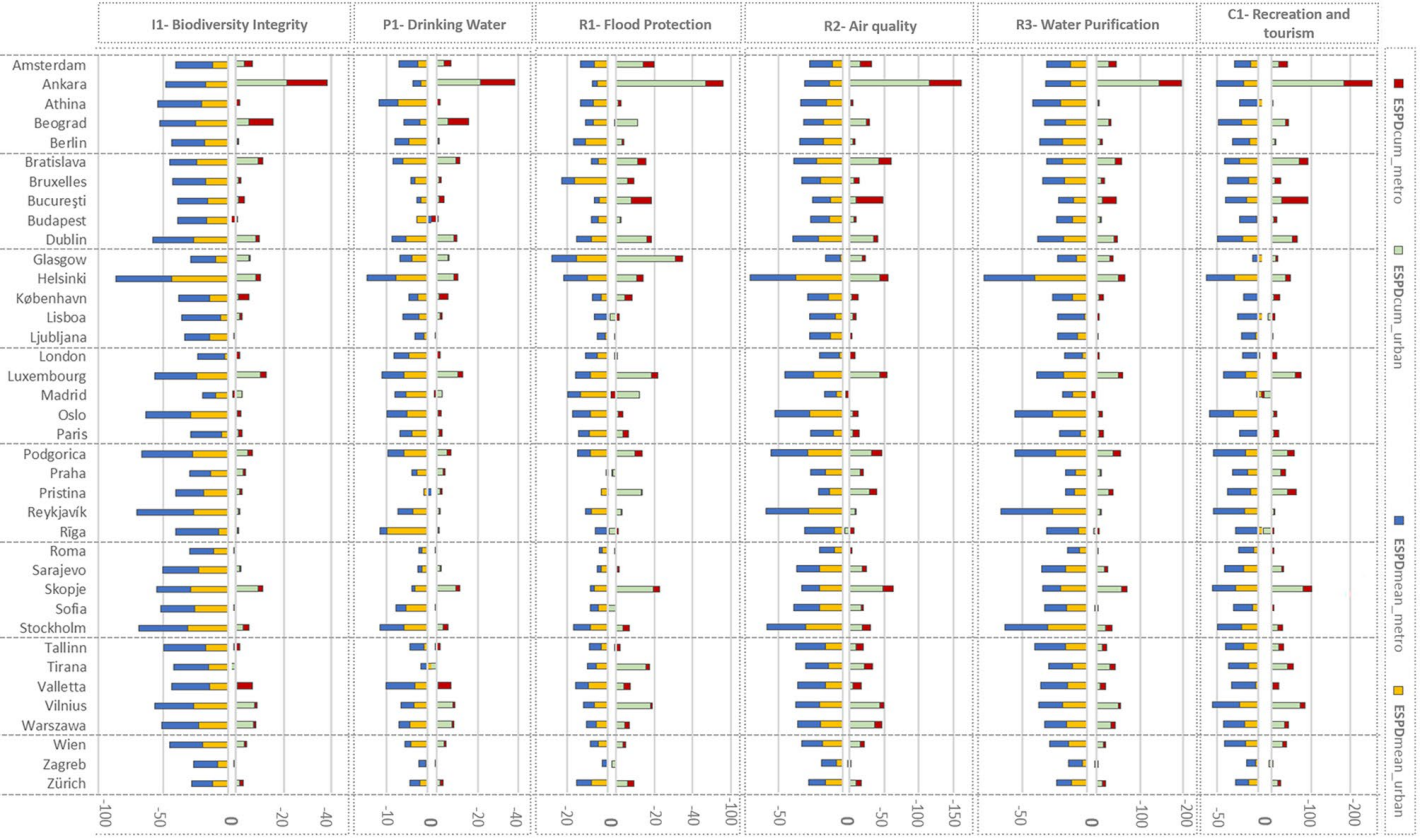
ESPDcum and ESPDmean records of all ECMA for six ES, comparing UA data of 2012 and 2018 at metropolitan and urban core level. ECMA are displayed in alphabetical order.

Our results showed that the *ESPDcum* values in most cases were higher in urban than metropolitan levels for all six ES (see Fig. 7). Only the urban areas of Bucharest, Kopenhagen, London, and Valletta have faced less ESPD change than the respective metropolitan areas. Nevertheless, in specific ES, we could recognize outlying cases. For instance, the potential of *flood protection* (R1) has changed similarly for both urban and metropolitan levels in the case of Bucharest.

**Figure 8:**
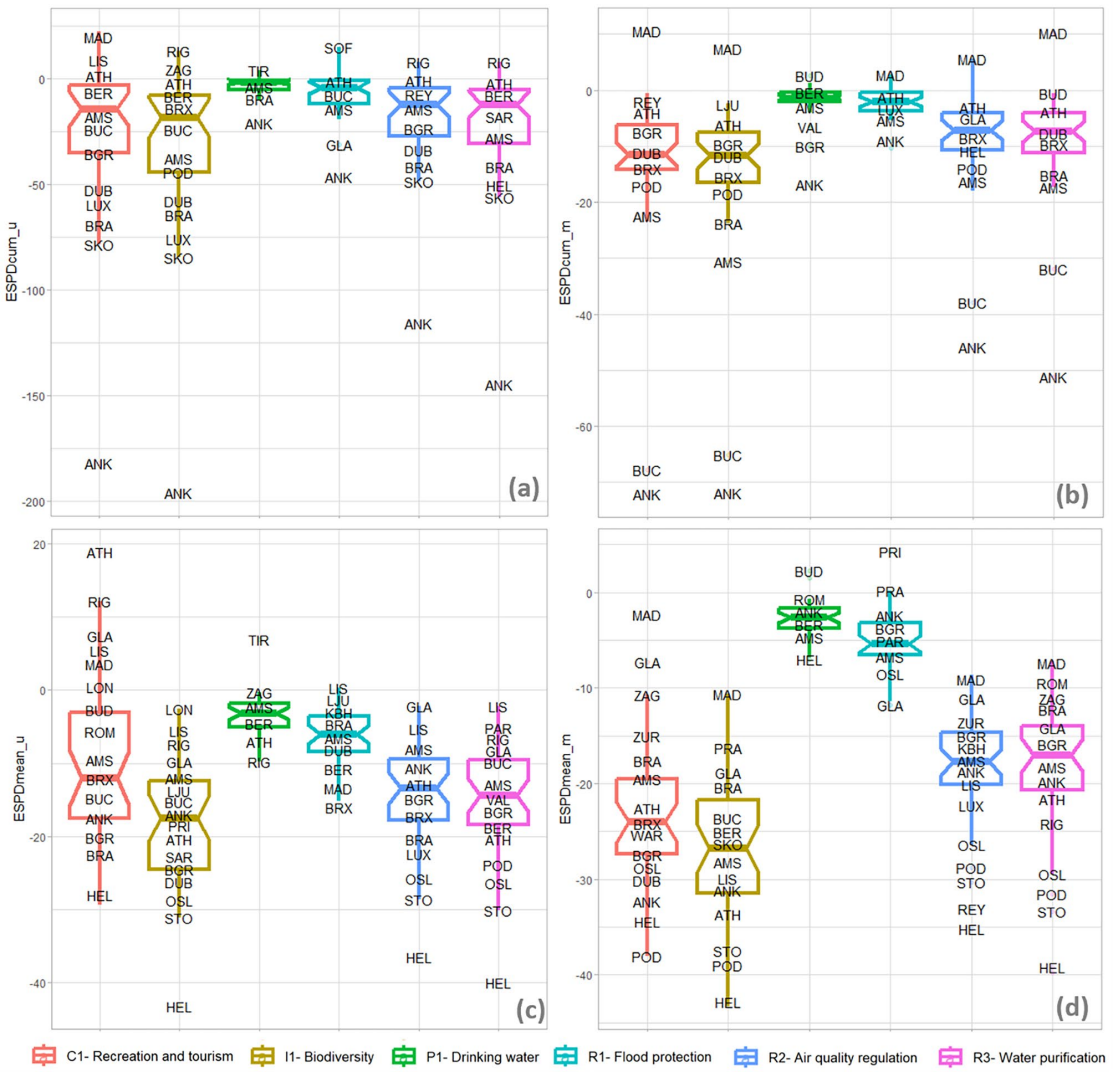
Boxplots of ESPDcum results of (**a**) urban and (**b**) metropolitan, and ESPDmean results of (**c**) urban and (**d**) metropolitan level, where: *AMS* Amsterdam, *ANK* Ankara, *ATH* Athina, *BGR* Beograd, *BER* Berlin, *BRA* Bratislava, *BRX* Bruxelles, *BUC* Bucharesti, *BUD* Budapest, *DUB* Dublin, *GLA* Glasgow, *HEL* Helsinki, *KBH* København, *LIS* Lisboa, *LJU* Ljubljana, *LON* London, *LUX* Luxembourg, *MAD* Madrid, *OSL* Oslo, *PAR* Paris, *POD* Podgorica, *PRA* Praha, *PRI* Pristina, *REY* Reykjavík, *RIG* Rīga, *ROM* Roma, *SAR* Sarajevo, *SKO* Skopje, *SOF* Sofia, *STO* Stockholm, *TAL* Tallinn, TIR Tirana, *VAL* Valletta, *VIL* Vilnius, *WAR* Warszawa, *WIE* Wien, *ZAG* Zagreb, *ZUR*  Zürich.

Ankara experienced the most significant change between 2012 and 2018 at the urban level for all ES types. This result is aligned with the information delivered in Fig. 6, where most cases of change in Ankara were inside the urban core. While at the metropolitan level, Ankara headed the ranking of *ESPDcum* values only for *biodiversity integrity* (I1), *drinking water provision* (P1), and *water purification* (R3). The highest potential reduction in *flood protection* (R1), *air quality* (R2), and *recreation and tourism* (C1) at the metropolitan level was recorded in Bucharest.

In contrast to *ESPDcum* values, the *ESPDmean* results were more evenly distributed between the urban and metropolitan contexts. Nevertheless, differences were still recognizable. For example, there was a considerable difference between the urban and metropolitan *ESPDmean* values of *drinking water* (P1) and *recreation and tourism* (C1). While the former ecosystem service potential (P1) was affected the highest at the urban level, the latter (C1) was reduced mostly at the metropolitan scale.

While Ankara was the case facing the highest total *ESPDcum*, Helsinki led the ranking of average ES reduction (*ESPDmean*), followed by other Nordic cities like Reykjavik, Stockholm, and Oslo (Fig. 7). It is worth mentioning that cities experiencing the highest average of ES potential reduction (*ESPDmean*) were among the ECMA with the highest current *ESPcum* (see Fig. 5). Nevertheless, cases like Ljubljana represented successful cases where the existing high ES potentials (ESPcum) were safeguarded by the minimal impact (ESPDmean) of LULC change on ES provision potential between 2012 and 2018.

ES potential for *drinking water provision* (P1) and *flood protection* (R1) were the least affected by LULC change (see Fig. 8b,d). At the same time, the average changes in the potential for *drinking water provision* (P1) and *flood protection* (R1) were remarkably higher at metropolitan levels than at urban ones (see Fig. 8a,c). The potential of *recreation and tourism* (C1) was the only ES that experienced less degradation at the urban level compared to the metropolitan one. On the other hand, the alterations at both urban and metropolitan scales affected the ES potential for *biodiversity integrity* (I1). Overall, while the cumulative ES potential degradation (ESPDcum) was higher at the urban level (see Fig. 8a,c), the *ESPDmean* was more intense at the metropolitan level (see Fig. 8b,d). This implies that the artificialisation was more frequent in urban and sub-urban zones but more impactful in rural areas.

#### ESP dynamics of ECMA between 2006–2012 and 2012–2018

**Figure 9:**
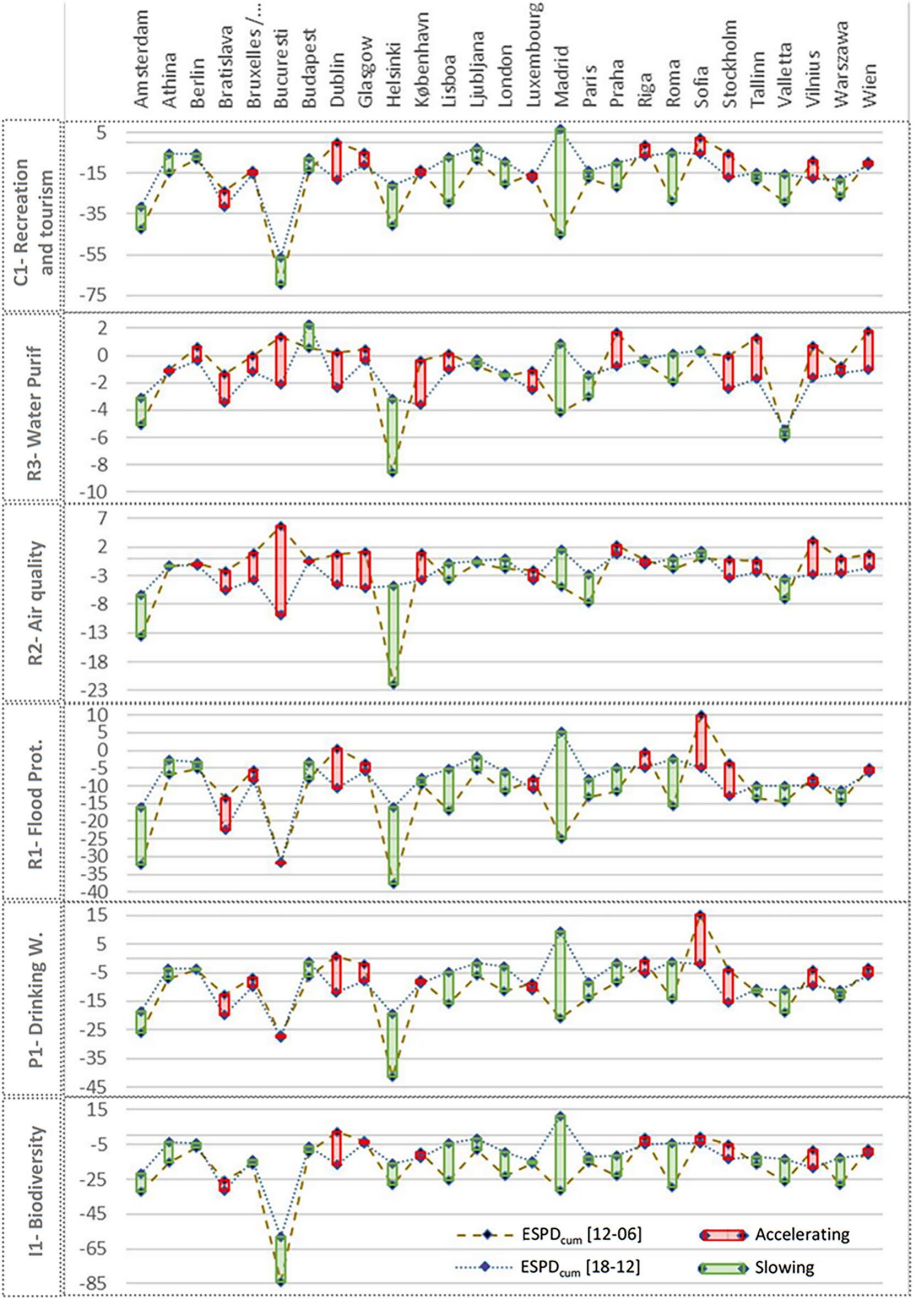
Change dynamics of ESPDcum between 2006 and 2012 (dashed line) and 2012–2018 (dotted line) for six ES. The vertical bar connecting two record points indicates the difference between the ESP dynamics of two periods (2006–2012 and 2012–2018). The color indicates the trend of accelerating ESP decrease (red) or a slowing down trend of ESP reduction (green).

For the 27 metropolitan areas included in this stage of the analysis, the ESP deterioration rates (*ESPDcum*) have been reduced by 34% between two periods (2006–2012 and 2012–2018) when considering all 27 cases combined (see Supplementary Dataset 3). This can be regarded as a positive result, indicating a relative slowing down in the declining trends of ecosystem services potential. However, among 27 ECMA, there were both accelerating (red) and slowing (green) cases (see Fig. 9).

While most of ECMA recorded a slowing down of ESP decline for *biodiversity integrity* (I1), *drinking water* (P1), *flood protection* (R1), and *recreation and tourism* (C1), 16 out of 27 recorded acceleration of ESP reduction rates regarding *air quality* (R2) and *water purification* (R3). On the other hand, while the ESP declining trends of most ECMA varied between accelerating and slowing depending on the ES type, there were ECMA cases where the ESP decline trends of which were constantly accelerating (like Bratislava, Dublin, Glasgow, Stockholm, Vilnius, and Wien) or slowing down (like Madrid, Helsinki, Rome, Amsterdam, Valleta, etc.) for all ES types.

### Correlating ESP dynamics to socio-economic and environmental factors

We correlated ESP, ESPDcum, and ESPDi results to socio-economic and environmental factors like population density, population growth, urban expansion, metropolitan GDP per capita, latitude, yearly sunny hours, and average temperature. In the following, we present the most significant correlations, while the remaining are included in the supplementary materials.

**Figure 10:**
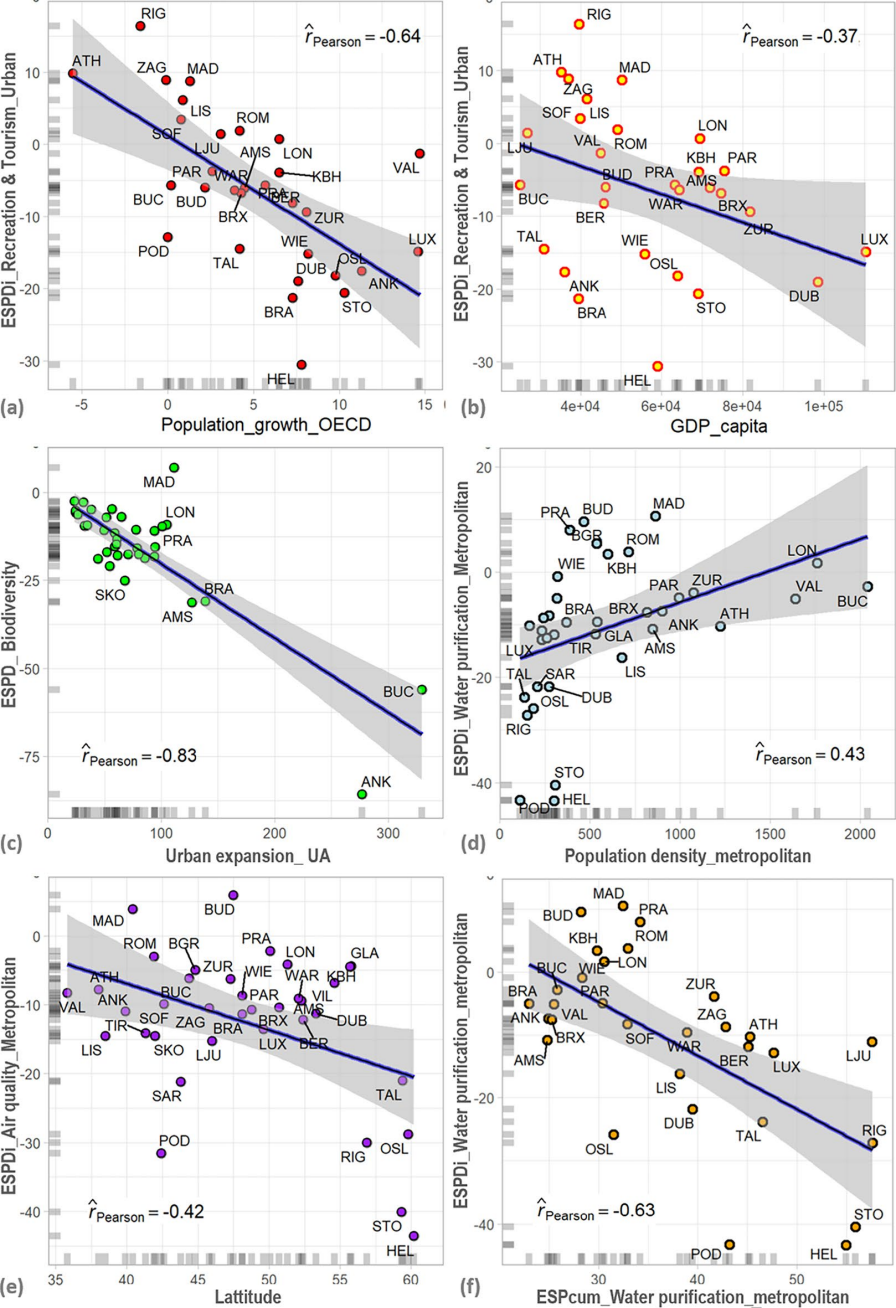
Correlation of ESPDcum (I1_Biodiversity) results to (**a**) Population growth ratio, (***b***) Urban expansion ratio, and correlation the ESP (I1_Biodiversity) results at urban scale to (***c***) yearly average temperature, (***e***) population density, and the results at metropolitan level to (***d***) yearly average temperature, (f) population density, where: *AMS* Amsterdam, *ANK* Ankara, *ATH* Athina, *BGR* Beograd, *BER* Berlin, *BRA* Bratislava, *BRX* Bruxelles, *BUC* Bucharest, *BUD* Budapest, *DUB* Dublin, *GLA* Glasgow, *HEL* Helsinki, *KBH* København, *LIS* Lisboa, *LJU* Ljubljana, *LON* London, *LUX* Luxembourg, *MAD* Madrid, *OSL*  Oslo, *PAR* Paris, *POD* Podgorica, *PRA*  Praha, *RIG* Rīga, *ROM* Roma, *SAR* Sarajevo, *SKO* Skopje, *SOF* Sofia, *STO* Stockholm, *TAL* Tallinn, *TIR* Tirana, *VAL* Valletta, *VIL* Vilnius, *WAR* Warszawa, *WIE* Wien, *ZAG* Zagreb, *ZUR* Zürich.

The highest correlation (negative) trendline was between the urban growth ratio, ESPD biodiversity, and recreational results (r_Pearson_ = − 0.83, see Fig. 10c and Fig. S4). The lowest correlation between urban expansion and ESPD is recorded for drinking water ES (r_Pearson_ = − 0.51, Fig. S4). Population density based on OECD data has a positive correlation (r_Pearson_ = 0.43, see Fig. 10d) to the ESPDi of water purification. A similar but lower positive correlation trend exists between urban population density and other ES types (see Fig. S8). This implies that the higher the urban population density, the less ESP reduction there will be. A similar but lower negative correlation resulted between urban GDP per capita (World Bank data) and ESPDi recreational and tourism (r_Pearson_ = − 0.37, see Fig. 10b). This implies that the higher the GDP per capita, the higher the ESP reduction. On the other hand, the urban population growth rates negatively correlate to the ESPDi recreation and tourism (r_Pearson_ = − 0.64, see Fig. 10a). Similar but lower negative correlations exist between urban population growth and other ES types (see Fig. S6). This implies that the higher the urban population growth, the higher the ESP reduction, which aligns with our initial hypothesis.

Yet, the demographical dynamics correlate less to the ESP reduction than the urban expansion (compare Figs. S2 and S6). In other words, we can conclude that the ESP reduction can be correlated more with urban development processes than demographic changes. Nevertheless, specific differences between cases can be drawn. For instance, Ankara, Helsinki, and Podgorica recorded relatively higher ratios of ESP reduction to urban growth rates (see Fig. 10c), implying an urban transformation trend of high impact on ecosystem services potentials, aligning well with the results on *ESPDmean* (see Fig. 7c,d). On the other hand, the urban expansion trends in cases like Madrid, London, and Prague had relatively less impact on ESP dynamics (see Fig. 10c). Despite the high population growth rates, cities with low ESPDi rates represent successful decision-making during land allocation for new urban developments or densifying the existing building stock like Valleta, London, and Luxemburg (see Fig. 10a).

Zagreb was the most durable case with the minimum change of population growth ratio and *ESPDcum* (I1-Biodiversity) value between 2012 and 2018. Even though Athens experienced a significant decrease in population (− 5.6%) in the same period, there was a reduction in ESPDcum by − 5.8. This implies that between 2012 and 2018, urban expansion continued despite the population shrinkage in Athens. Further details on the urban expansion and urban population growth rates are provided in the supplementary information (see Tables S3 and S4).

We also found a reasonable correlation between ESPDi results and environmental factors, like average temperature, yearly sunny hours, and latitude. The highest negative correlation is recorded between latitude and metropolitan air quality (r_Pearson_ = − 0.42, see Fig. 10e). Yearly sunny hours are the least correlated environmental factor to the impact of ESP reduction (ESPDi). Meanwhile, the average temperature draws a lower positive correlation trend, opposing the latitude trend (see Figs. S3 and S4). We also found that the current ESP (ESPcum) significantly correlates to the ESPDi regarding water purification (r_Pearson_ = − 0.63, see Fig. 10f). It implies that the ECMA with the highest current ES potential, have experienced the ESP reduction of the highest impact, like Stockholm, Helsinki, Podgorica, and Oslo (see Fig. 10f).

## Discussion

The disparities among ECMA regarding ESP and ESPD results provide ground to better understand other potential indicators of ESP dynamics at the local scale. This is imperative to identify constructive suggestions for planning actors and enable more case-sensitive and sustainable management of ongoing metropolitan development processes. On the other hand, we should admit that our study focused on the quantitative translation of LULC change to ES potential and did not consider the local scale qualitative values as perceived by people at each ECMA. For example, the low recreational and tourism ESP results of Reykjavik (Fig. 5f), at first look, may sound contradictory to the beauties of the Arctic sceneries. However, our results do not aim to conclude any qualitative evaluation, and we acknowledge that each ECMA bears intangible values that are not within this study's scope.

We argue that the variations between ECMA in ESP dynamics at the metropolitan level are primarily due to geographical location and the related environmental conditions. The effects of projected global warming on the ESP dynamics at the metropolitan scale will be more evident soon, as the consequences of climate change have intensified in recent decades. In contrast, the ES potential at the urban level is affected mainly by the socio-political approach in urban land use, management, and planning decision-making processes. Our study highlights specific ECMA cases whose ESP results are sensitive to the respective geographical, cultural, and socio-political backgrounds.

First, the metropolitan-level cumulative ESP results (Fig. 5) are related to the geographical location and the local climate. For example, the low *ESPcum* results of Reykjavik are primarily due to the inherited open spaces with little or no vegetation (beaches, dunes, bare rocks, glaciers), which dominate the metropolitan surface areas surrounding the most significant urban center of Iceland. Furthermore, local scholars have shown that the LULC alteration in Iceland continues due to rising temperatures, volcano events, and land use change^38^. This process is still accelerating despite the local community's reforestation efforts and the “green waves” during the twentieth century^39^.

Furthermore, the high ESP reduction (*ESPDmean*) in Fennoscandian (Helsinki, Oslo, and Stockholm) metropolitan areas (see Fig. 7) is because 54% of altered surfaces between 2012 and 2018 were previously covered by forests (41%), pastures (11%), and herbaceous vegetation associations (2%) (see Supplementary Data). These areas were mainly converted to mineral extraction and dump sites (25%), construction sites (20%), industrial, commercial, public, military, and private units (18%), and discontinuous dense urban fabric (12%). Our results suggest that human activity is the primary driver of this change. Nonetheless, an increase in the impact of climate change on ESP dynamics is expected, as implied by the findings of local researchers. They reveal a continuous reduction in the normalized difference vegetation index (NDVI as an indicator of vegetation cover healthiness) values and have linked it to less cold winter season^40^ and extreme weather conditions in summer^41^. Recent large-scale wildfire events in 2019 across Scandinavia are related to extremely dry conditions^42^ and significantly impact land cover change and the respective ES potential.

On the other hand, the ESP dynamics at the urban level are primarily affected by the socio-political approaches in metropolitan/urban planning. According to previous studies, the much-advocated compact city concept was not advantageous for urban ES provisioning^22^. This statement is aligned with our results, where less compact urban centers with lower population densities, like the Scandinavian metropolitan areas, record higher levels of *ESPcum* (see Fig. 5 and Fig. 10e). Similarly, reduced ESP values belong to ECMA located in post-socialist countries of South-eastern Europe where the political changes of the post-90 s boosted extensive “anarchic” urban sprawl in the wildland-urban interface of capital metropolitan areas^43,44^, and the post-2000s densification in the city centers^45^. Novel approaches in urban planning promote urban green infrastructure as an effective method to better balance the negative impacts of urban sprawl processes^46^, which would remarkably contribute to the ESP at the metropolitan and urban levels.

At first look, urban expansion generates several challenges for LULC management. However, ecological function restoration can bring some opportunities to plan healthier and more resilient metropolitan areas^8^. Thus, decision-making processes in urban planning and management must consider the ES potential of the metropolitan lands and their rapidly changing trends, as our results have shown. The ES provision is improvable in critical cases where restoration of ecological functions is possible. Thus, society must develop methods and procedures to quantify, monitor, and preserve the existing metropolitan surfaces of high ESP for further ecological enhancement. This is vital when political awareness is at its highest level ever at both the global and EU levels.

At the global scale, the 17 Sustainable Development Goals (SDGs) set by the United Nations include 91 out of 169 targets that require action regarding urban ecosystem management^47^. Most ES are advocated to have cross-target implications to SDGs led by food and water provision, biodiversity maintenance, and carbon storage & sequestration, which are perceived to simultaneously contribute to more than 14 SDG targets^48^. Similarly, at the European level, following the Habitats Directive, in the summer of 2022, the EU proposed the European Nature Restoration Law (ENRL) to ensure the safeguarding and improvement of existing European natural areas^49^. Among many objectives, ENRL targets a zero net loss of urban green spaces in every EU city, town, and suburb by 2030 and a minimum of 10% tree canopy cover in every EU city, town, and suburb by 2050^50^. We are hopeful that a dedicated implementation of ENRL at the metropolitan level will significantly impact the improvement of ESP across ECMA.

However, downscaling ENRL and mainstreaming it to local-level policies may not be as straightforward as it sounds, but case-based application mechanisms will be required. For instance, policymakers governing ECMA should revise the ENRL's targeted measures and goals by adapting them to their specific cities while considering the socio-economic and environmental conditions. The adaptation instruments must be rooted in participatory decision-making principles by involving representatives from all stakeholder groups. Mechanisms like stakeholders’ roundtables, in-depth interviews with focus groups, public meetings, opinion polls, and voting are vital to involve the public in future development goals and secure a context-sensitive adaptation of ENRL. It is also essential that these events are repeated at certain time intervals as an instrument to ensure a long-run implementation of ENRL. This will facilitate setting specific threshold values for the status and change of the metropolitan natural systems and ensuring the improvement of ES potential via natural systems enhancement.

Our results suggest urban expansion remains the critical challenge in safeguarding the existing natural systems and the related metropolitan ES potential. At the EU level, where 75% of the buildings are for residential use^51,52^, metropolitan area managers must find innovative solutions to the housing necessities of the rising urban population while achieving zero loss of existing ES. More than 11 million housing units are reported unoccupied in the EU^51^. Innovating schemes for reactivating the unused housing stock could minimize the new land consumption for new residential needs. This is urgent for cities like Bucharest and Athens, which are facing EP potential reduction despite the shrinking population, as reported in this study (see Fig. 10b). However, reactivating the existing housing stock can not be the solution alone; instead, it is one among many other measures to be taken by responsible governments. For example, the development of offsetting policies, where cities that develop new built-up areas must create or improve natural areas elsewhere, could contribute towards zero-loss of ES potential.

Another highlight is the relation between landscape fragmentation/ connectivity and ES provision. Recent literature has shown that abundant green and water surfaces alone are insufficient for an effective blue-green infrastructure in metropolitan regions^53^. Since various ecosystem services rely on materials and organisms flow across landscapes^54^, scholars have advocated reconsidering landscape connectivity through the scope of ES provision^55^. This relation expands the importance of habitat fragmentation from a merely ecological concern into a socio-ecological urge^56^. More concretely, the transportation network is among the main fragmenting agents in the metropolitan regions, causing severe interruptions in the natural flows of materials and ES provision^57^. Thus, transportation network planners and metropolitan area managers must consider landscape connectivity indispensable while designing new transportation routes and renovating the existing ones.

In this study, we relied on UA data, which provide relatively coarse-scale information based on LULC properties. This information can be considered appropriate for metropolitan-level studies but may fall short for site-scale assessment of urban ES. For instance, the presence of smaller natural elements like urban trees^58^ and private/public allotment gardens is indispensable in providing urban ecosystem services^59^. However, we are intentionally satisfied with UA data here since it is a pan-European product generated via a harmonized methodology. Furthermore, we acknowledge that assessing diverse ES relying on LULC data alone may be insufficient to detect socio-ecological dynamics at the local scale since the provision capacities of specific ES (ex. flood protection, drinking water storage, etc.) may depend on other factors like the demographical properties (ex. population density) of the study area. However, ES mapping approaches that rely directly on LULC information are accepted as appropriate for coarse-scale studies in which the focus is on estimating ES potential instead of quantifying the ES supply^60^. Future research focusing on specific metropolitan capital cities should consider integrating the information about the fine-scale natural elements and other demographical aspects in the ESP assessment.

Despite the limitations at the local scale, our study provides for the first time a pan-European level (including non-EU countries) characterization of metropolitan ESP dynamics. Previous studies have analyzed specific examples from EU metropolitan areas like Barcelona, Berlin, Helsinki, Salzburg, and Stockholm^20,21^, which makes it difficult to draw general conclusions and to identify outliers. About one decade ago, Larondelle et al. presented the only large-scale analysis comparing the ES capacities of large metropolitan areas within EU-27 member states^22^. However, these studies have relied on the data of 2006, the latest updated information in those days, and without any temporal analysis on the ES provision change considering more than one period’s records. In this work, we advanced the knowledge inherited from previous studies by enabling a spatiotemporal analysis considering a time span of 12 years (2006, 2012, 2018) relying on UA data, which is finer in resolution than CLC data. Furthermore, we expand the geographical scope of previous studies, which included cities only from EU member states, by including the ES provision dynamics in developing metropolitan areas in the remaining European countries (non-EU). The method we presented here resulted in an effective and rapid procedure to compare the spatiotemporal ES potential dynamics across ECMA using fine-scale geospatial data at a pan-European level.

Eventually, our results and the promising international agendas on restoring metropolitan natural systems will fall short if not accepted and implemented at local levels. Diverse stakeholders are responsible for safeguarding and improving the metropolitan ES, including decision-makers, local businesses, citizens, and even visitors and tourists. This understanding is the basis of establishing novel and context-sensitive mechanisms for safeguarding and enhancing the natural systems at local levels. Decision-making actors must be especially aware that any decision made for a new land use allocation directly impacts ES potential and will be irreversible, at least for several decades. Otherwise, unreflective decisions may have long-term harmful impacts on the ES potential and many other ecological functioning distributions across the metropolitan areas throughout Europe.

## Conclusions

We investigated the ES potential dynamics among 38 European capital metropolitan areas based on the LULC information by UA data and the Expert Matrix method. This work is the first comparative pan-European study relying on a unified dataset on LULC change. Our analysis of the six most relevant urban ES showed that *biodiversity integrity* (I1), *recreation and tourism* (C1), *air quality* (R2), and *water purification* (R3) are the ES types affected the most by LULC change, while *drinking water* (P1) and *flood protection* (R1) are the least sensitive ES by LULC alteration. At the case level, Fennoscandian cities record the highest existing ESP. However, the ESP dynamics analysis has shown that they have experienced the highest impact of ESP reduction between 2006, 2012, and 2018, implying unstable future scenarios for these metropolitan regions. We also discussed the reasons behind this fact and argued that they should be investigated further in-depth by future studies. Reasonably, most of this change is human induced due to new land use allocation. However, potential implications of climate change consequences at the metropolitan level must be investigated as triggered by our results regarding Nordic cities. Methodically, we are satisfied with the periodic time span of 6 years due to the wide range of UA nomenclature. However, future studies should consider investigating via finer temporal scales through LULC maps generated from available remotely sensed satellite images. Despite these limitations, our study showed that the ES potential of European capital metropolitan areas has significantly reduced in recent years. Consequently, we urge that urban management and planning stakeholders must reflect on the consequences of their LULC alteration actions on the ES provision potential within the metropolitan areas under their responsibility.

### Supplementary Information

Supplementary Information.

## Data Availability

The raw data used in this study are available from Copernicus, Land Monitoring Service by European Union via the following link, https://land.copernicus.eu/en/products/urban-atlas. The datasets generated and analyzed during the current study are archived in the Zenodo public repository and are available via the following 10.5281/zenodo.10793967.
