# Supplementary material for: Ecosystem services potential is declining across European capital metropolitan areas
Source: Sci Rep. 2024 Apr 17;14:8903. doi: 10.1038/s41598-024-59333-8 (PMC11023948; doi:10.1038/s41598-024-59333-8)
Supplement: Supplementary file 1 — Supplementary Information. [file 41598_2024_59333_MOESM1_ESM.pdf]

Ecosystem Services Potential is Declining across European Capital Metropolitan Areas

Artan Hysa, Roland Löwe, Juergen Geist

Supplementary Information

Calculation of indicators on a real case

According to the UA data in 2012, the area was originally covered by forests, as shown in the orthophoto image of 2010. Only six years later, 97 hectares of these forest surfaces have been cleared to give the floor to new residential building construction (see orthophoto of 2018 in Figure 4). This directly affects the ESP of the respective patch, which drops from 80 to 10.

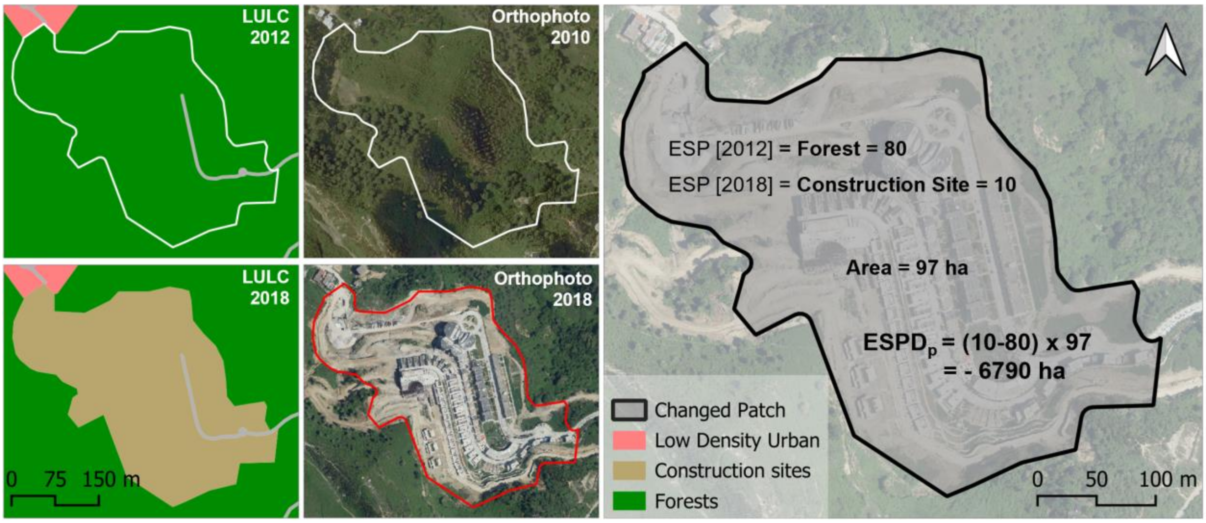

Figure S1. Identification of Patch and the ESP dynamics (I1-Biodiversity) based on LULC change. The selected patch is located on the southern outskirts of Tirana (Albania). The maps are authentic and produced by the first author using open access orthophoto imagery provided as WMS service by the Albanian National Authority for Geospatial Information (ASIG) via the ASIG geoportal (https://geoportal.asig.gov.al/geonetwork/srv/eng/catalog.search?auto=true#/metadata/4aa2f756-0bec-4a25-a1a8-662bda31ff4f). The land use information received from Urban Atlas data (Copernicus land monitoring service via https://land.copernicus.eu/en/products/urban-atlas). The maps are generated via open source software QGIS Desktop 3.24.0.

Correlation of ESP, ESPD, and ESPDi results to socio-economic and climate factors

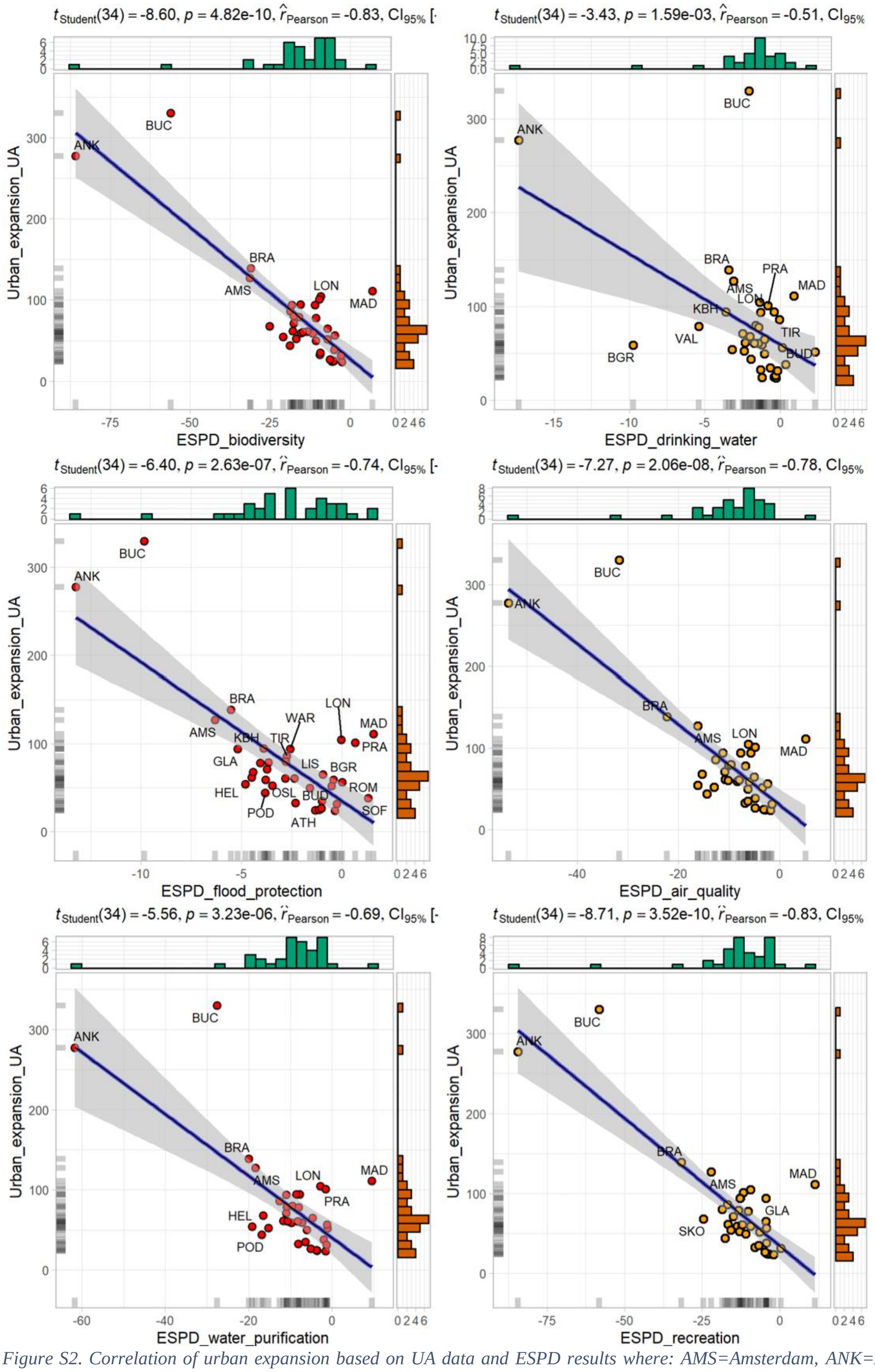

Figure S2. Correlation of urban expansion based on UA data and ESPD results where: AMS=Amsterdam, ANK=Ankara, ATH=Athina, BGR=Beograd, BER=Berlin, BRA=Bratislava, BRX=Bruxelles, BUC=Bucureşti, BUD=Budapest, DUB=Dublin, GLA=Glasgow, HEL=Helsinki, KBH=København, LIS=Lisboa, LJU=Ljubljana, LON=London,

LUX=Luxembourg, MAD=Madrid, OSL=Oslo, PAR=Paris, POD=Podgorica, PRA=Praha, RIG=Rīga, ROM=Roma, SAR=Sarajevo, SKO=Skopje, SOF=Sofia, STO=Stockholm, TAL=Tallinn, TIR=Tirana, VAL=Valletta, VIL=Vilnius, WAR=Warszawa, WIE=Wien, ZAG=Zagreb, ZUR=Zürich.

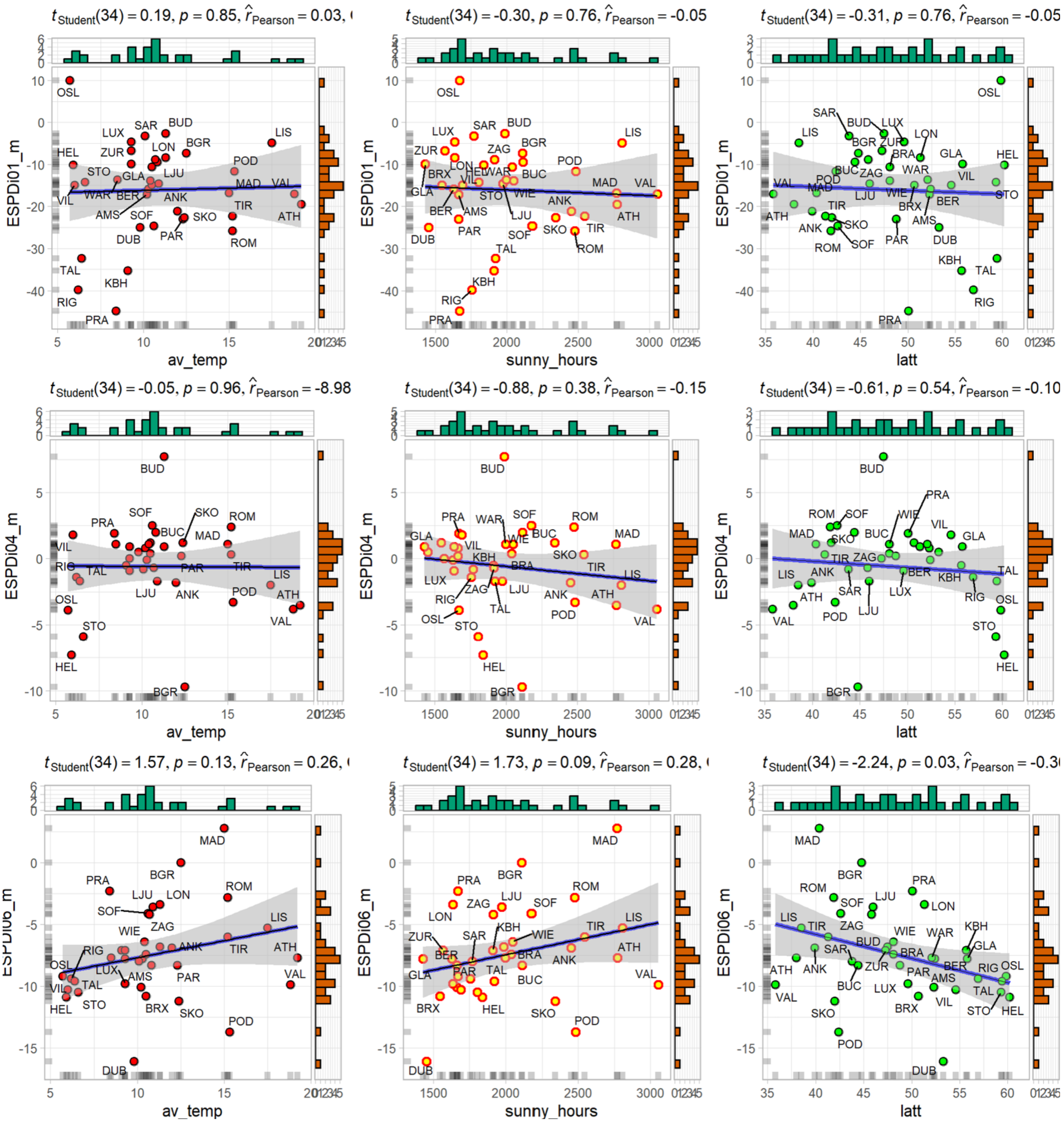

Figure S3. Correlation of ESPDi to environmental factors like average temperature, yearly sunny hours, and latitude, where: AMS=Amsterdam, ANK=Ankara, ATH=Athina, BGR=Beograd, BER=Berlin, BRA=Bratislava, BRX=Bruxelles, BUC=Bucureşti, BUD=Budapest, DUB=Dublin, GLA=Glasgow, HEL=Helsinki, KBH=København, LIS=Lisboa, LJU=Ljubljana, LON=London, LUX=Luxembourg, MAD=Madrid, OSL=Oslo, PAR=Paris, POD=Podgorica, PRA=Praha, RIG=Rīga, ROM=Roma, SAR=Sarajevo, SKO=Skopje, SOF=Sofia, STO=Stockholm, TAL=Tallinn, TIR=Tirana, VAL=Valletta, VIL=Vilnius, WAR=Warszawa, WIE=Wien, ZAG=Zagreb, ZUR=Zürich.

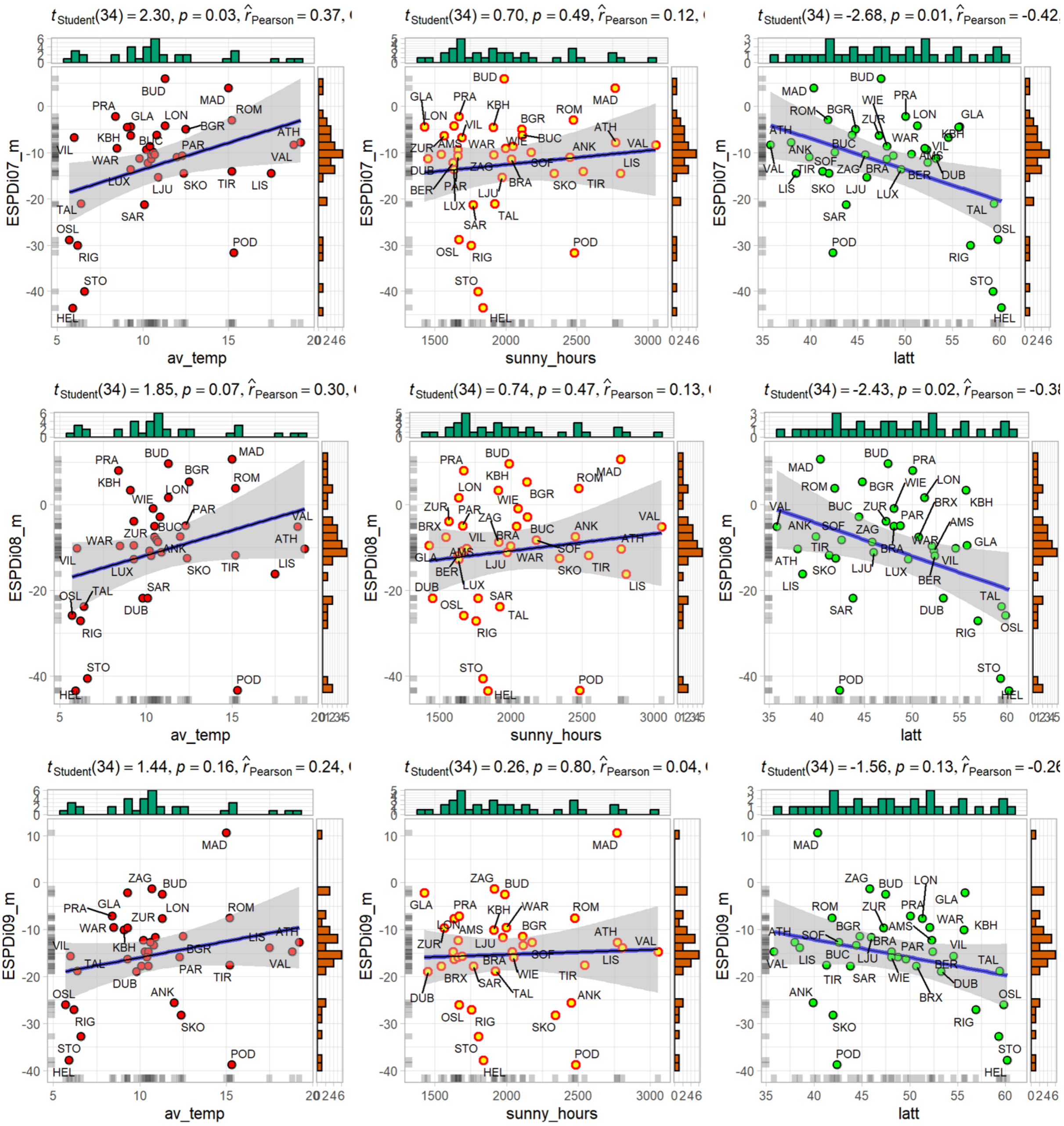

Figure S4. Correlation of ESPDi to environmental factors like average temperature, yearly sunny hours, and latitude, where: AMS=Amsterdam, ANK=Ankara, ATH=Athína, BGR=Beograd, BER=Berlin, BRA=Bratislava, BRX=Bruxelles, BUC=Bucureşti, BUD=Budapest, DUB=Dublin, GLA=Glasgow, HEL=Helsinki, KBH=København, LIS=Lisboa, LJU=Ljubljana, LON=London, LUX=Luxembourg, MAD=Madrid, OSL=Oslo, PAR=Paris, POD=Podgorica, PRA=Praha, RIG=Rīga, ROM=Roma, SAR=Sarajevo, SKO=Skopje, SOF=Sofia, STO=Stockholm, TAL=Tallinn, TIR=Tirana, VAL=Valletta, VIL=Vilnius, WAR=Warszawa, WIE=Wien, ZAG=Zagreb, ZUR=Zürich.

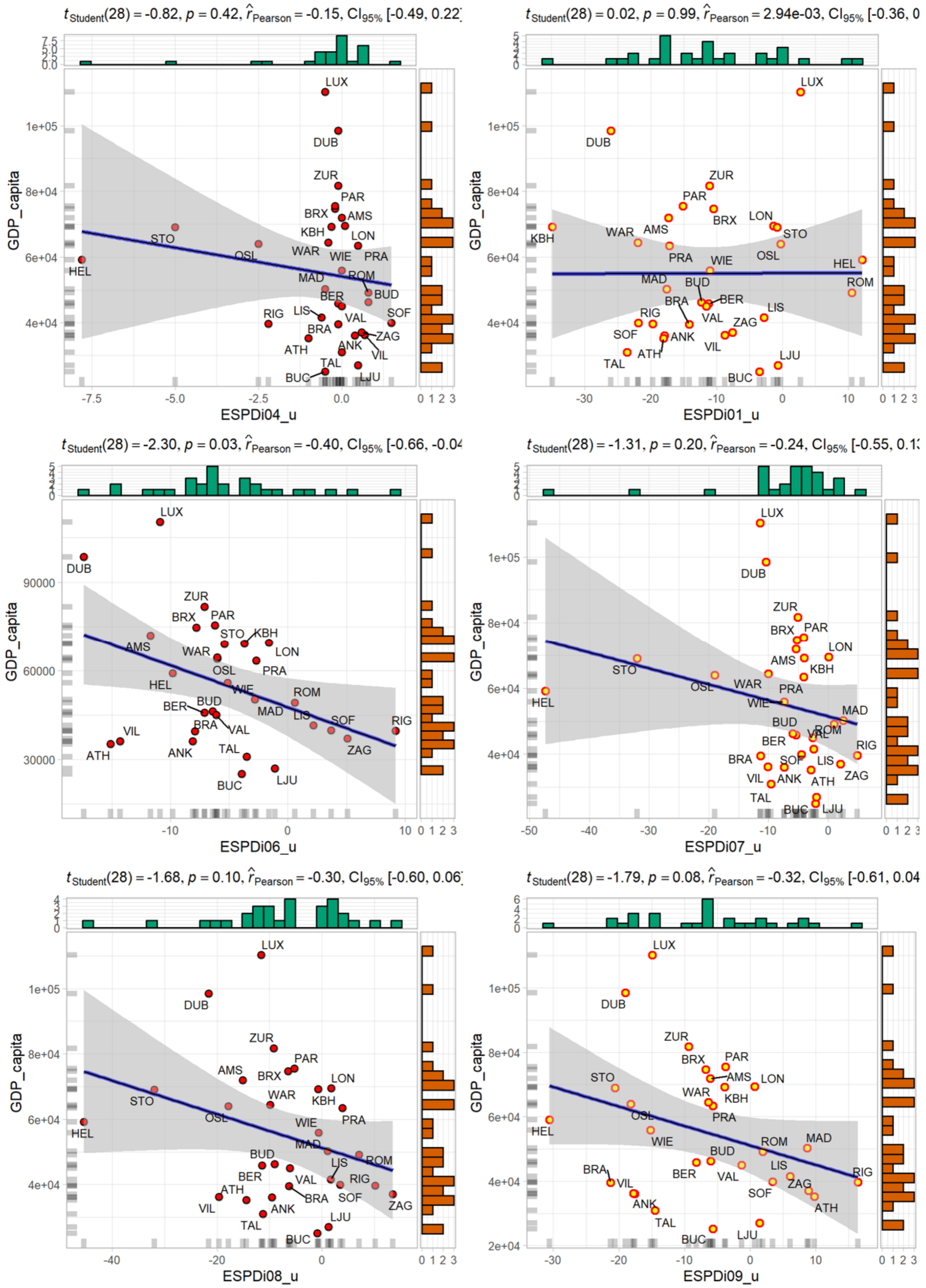

Figure S5. Correlation of ESPDi to metropolitan GDP per capita, where: AMS=Amsterdam, ANK=Ankara, ATH=Athina, BGR=Beograd, BER=Berlin, BRA=Bratislava, BRX=Bruxelles, BUC=Bucureşti, BUD=Budapest, DUB=Dublin, GLA=Glasgow, HEL=Helsinki, KBH=København, LIS=Lisboa, LJU=Ljubljana, LON=London, LUX=Luxembourg, MAD=Madrid, OSL=Oslo, PAR=Paris, POD=Podgorica, PRA=Praha, RIG=Rīga, ROM=Roma, SAR=Sarajevo, SKO=Skopje, SOF=Sofia, STO=Stockholm, TAL=Tallinn, TIR=Tirana, VAL=Valletta, VIL=Vilnius, WAR=Warszawa, WIE=Wien, ZAG=Zagreb, ZUR=Zürich.

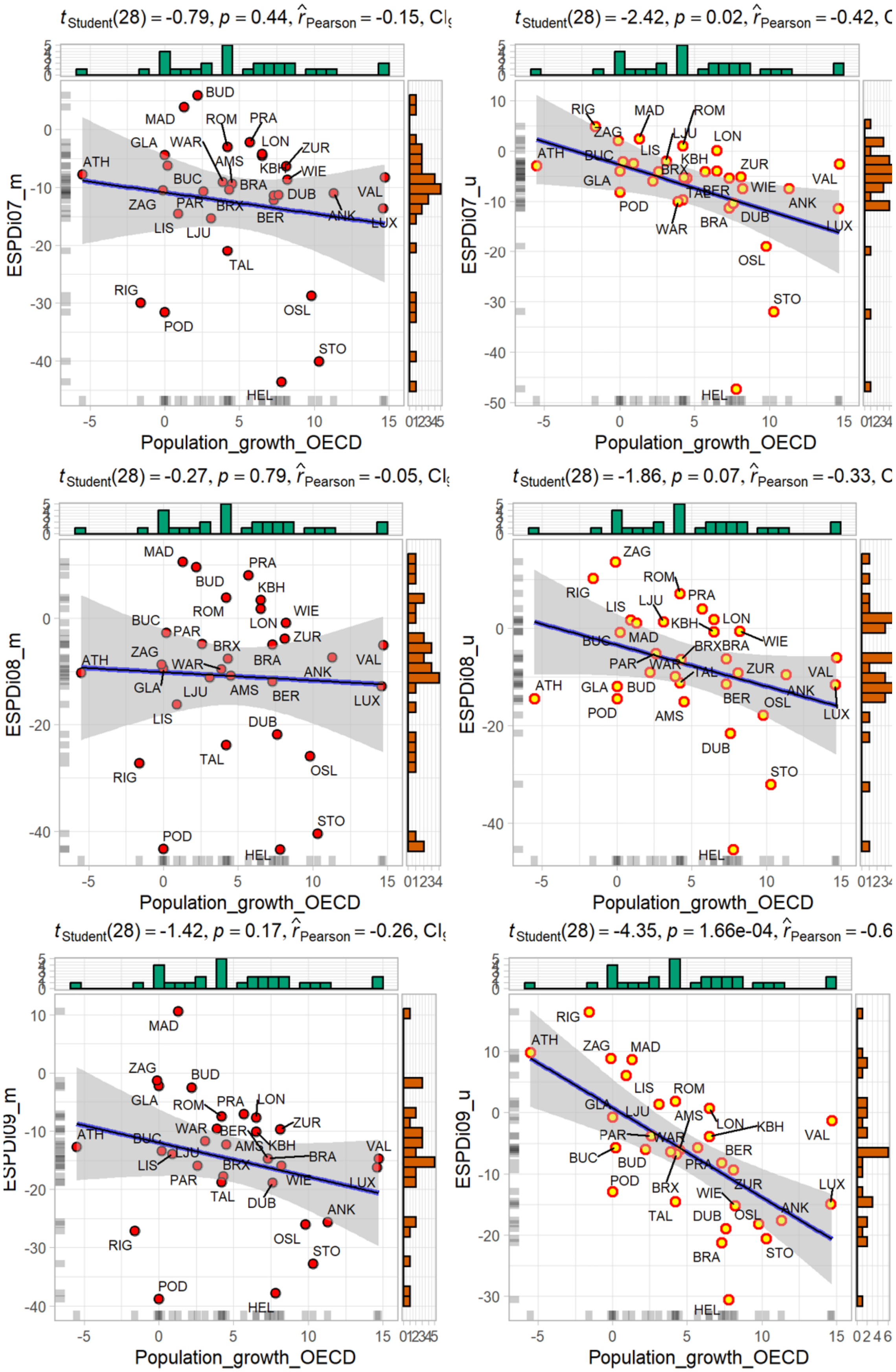

Figure S6. Correlation of ESPDi to metropolitan GDP per capita, where: AMS=Amsterdam, ANK=Ankara, ATH=Athina, BGR=Beograd, BER=Berlin, BRA=Bratislava, BRX=Bruxelles, BUC=Bucureşti, BUD=Budapest, DUB=Dublin, GLA=Glasgow, HEL=Helsinki, KBH=København, LIS=Lisboa, LJU=Ljubljana, LON=London, LUX=Luxembourg, MAD=Madrid, OSL=Oslo, PAR=Paris, POD=Podgorica, PRA=Praha, RIG=Rīga, ROM=Roma, SAR=Sarajevo, SKO=Skopje, SOF=Sofia, STO=Stockholm, TAL=Tallinn, TIR=Tirana, VAL=Valletta, VIL=Vilnius, WAR=Warszawa, WIE=Wien, ZAG=Zagreb, ZUR=Zürich.

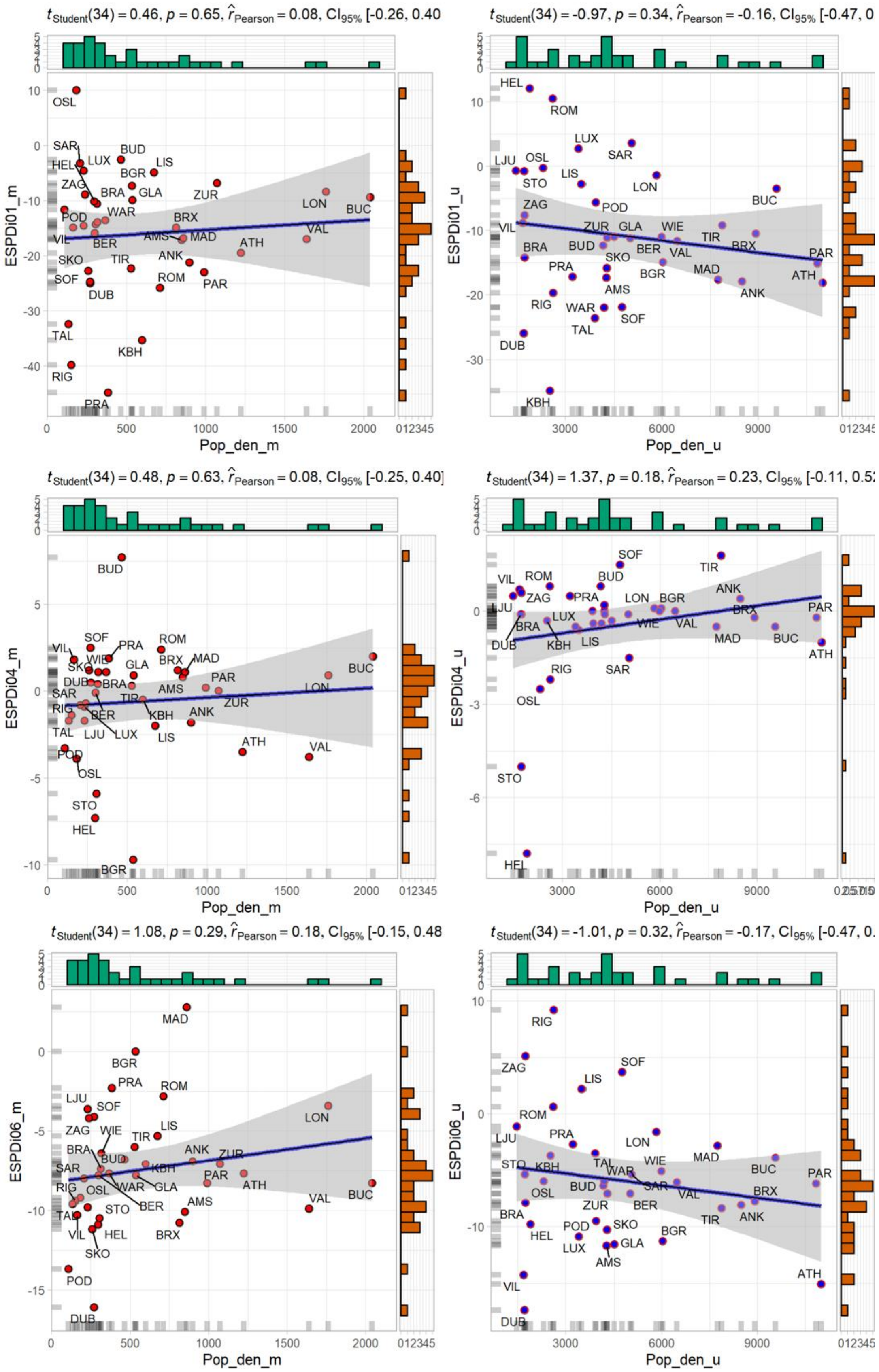

Figure S7. Correlation of ESPDi to population density, where: AMS=Amsterdam, ANK=Ankara, ATH=Athina, BGR=Beograd, BER=Berlin, BRA=Bratislava, BRX=Bruxelles, BUC=Bucureşti, BUD=Budapest, DUB=Dublin, GLA=Glasgow, HEL=Helsinki, KBH=København, LIS=Lisboa, LJU=Ljubljana, LON=London, LUX=Luxembourg, MAD=Madrid, OSL=Oslo, PAR=Paris, POD=Podgorica, PRA=Praha, RIG=Rīga, ROM=Roma, SAR=Sarajevo, SKO=Skopje, SOF=Sofia, STO=Stockholm, TAL=Tallinn, TIR=Tirana, VAL=Valletta, VIL=Vilnius, WAR=Warszawa, WIE=Wien, ZAG=Zagreb, ZUR=Zürich.

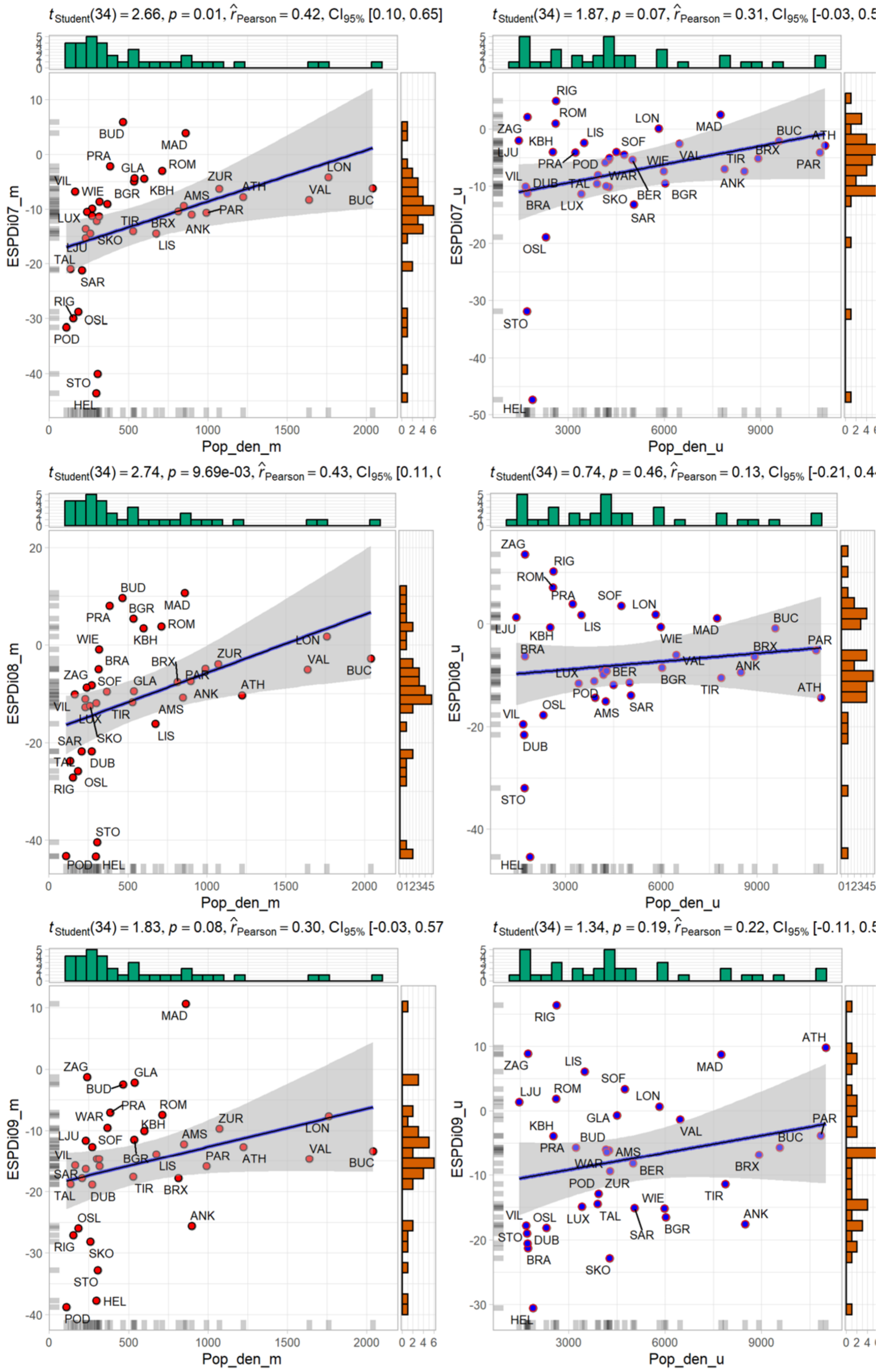

Figure S8. Correlation of ESPDi to population density, where: AMS=Amsterdam, ANK=Ankara, ATH=Athina, BGR=Beograd, BER=Berlin, BRA=Bratislava, BRX=Bruxelles, BUC=Bucureşti, BUD=Budapest, DUB=Dublin, GLA=Glasgow, HEL=Helsinki, KBH=København, LIS=Lisboa, LJU=Ljubljana, LON=London, LUX=Luxembourg, MAD=Madrid, OSL=Oslo, PAR=Paris, POD=Podgorica, PRA=Praha, RIG=Rīga, ROM=Roma, SAR=Sarajevo, SKO=Skopje, SOF=Sofia, STO=Stockholm, TAL=Tallinn, TIR=Tirana, VAL=Valletta, VIL=Vilnius, WAR=Warszawa, WIE=Wien, ZAG=Zagreb, ZUR=Zürich.

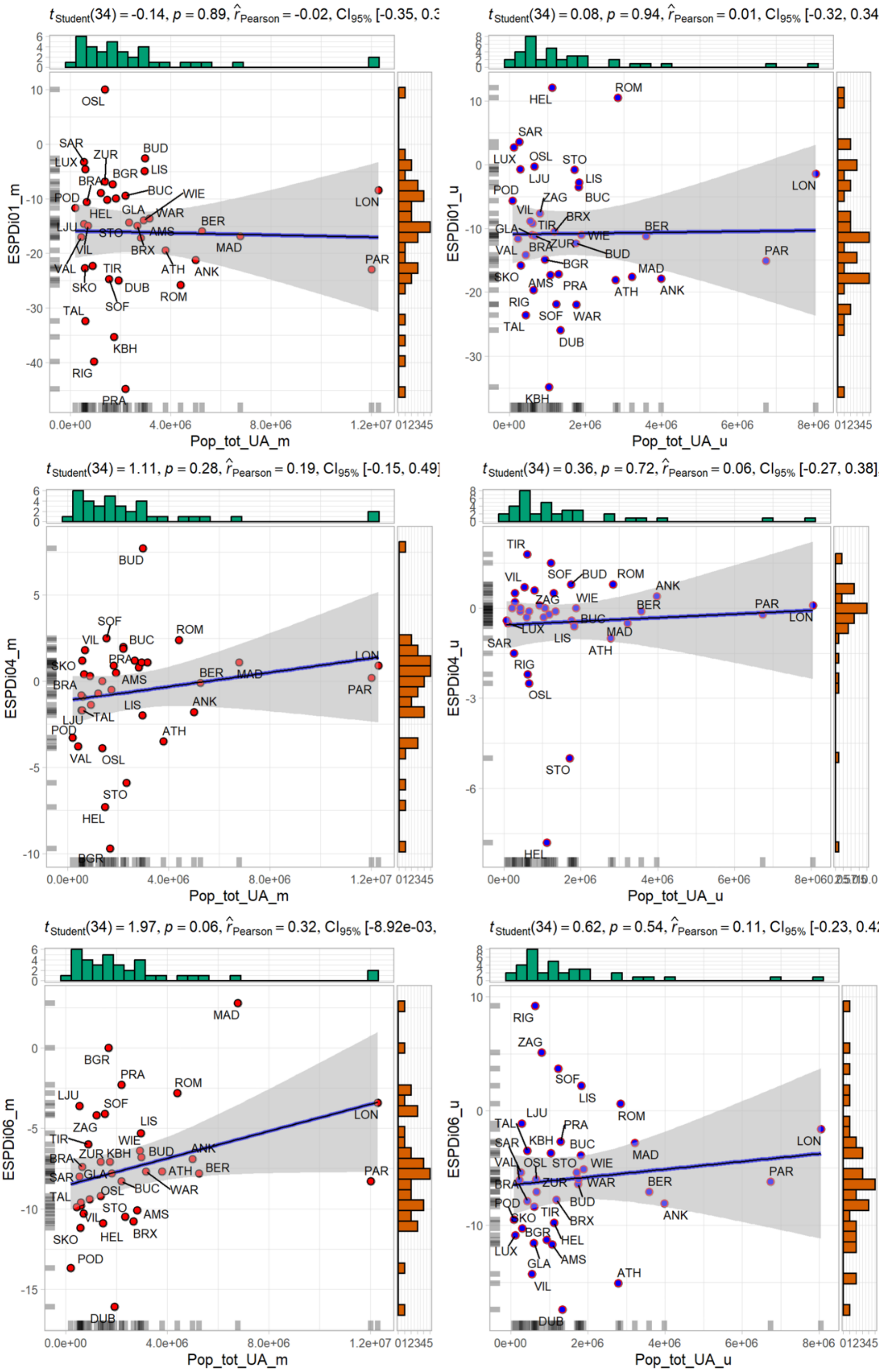

Figure S9. Correlation of ESPDi to total population, where: AMS=Amsterdam, ANK=Ankara, ATH=Athina, BGR=Beograd, BER=Berlin, BRA=Bratislava, BRX=Bruxelles, BUC=Bucureşti, BUD=Budapest, DUB=Dublin, GLA=Glasgow, HEL=Helsinki, KBH=København, LIS=Lisboa, LJU=Ljubljana, LON=London, LUX=Luxembourg, MAD=Madrid, OSL=Oslo, PAR=Paris, POD=Podgorica, PRA=Praha, RIG=Rīga, ROM=Roma, SAR=Sarajevo, SKO=Skopje, SOF=Sofia, STO=Stockholm, TAL=Tallinn, TIR=Tirana, VAL=Valletta, VIL=Vilnius, WAR=Warszawa, WIE=Wien, ZAG=Zagreb, ZUR=Zürich.

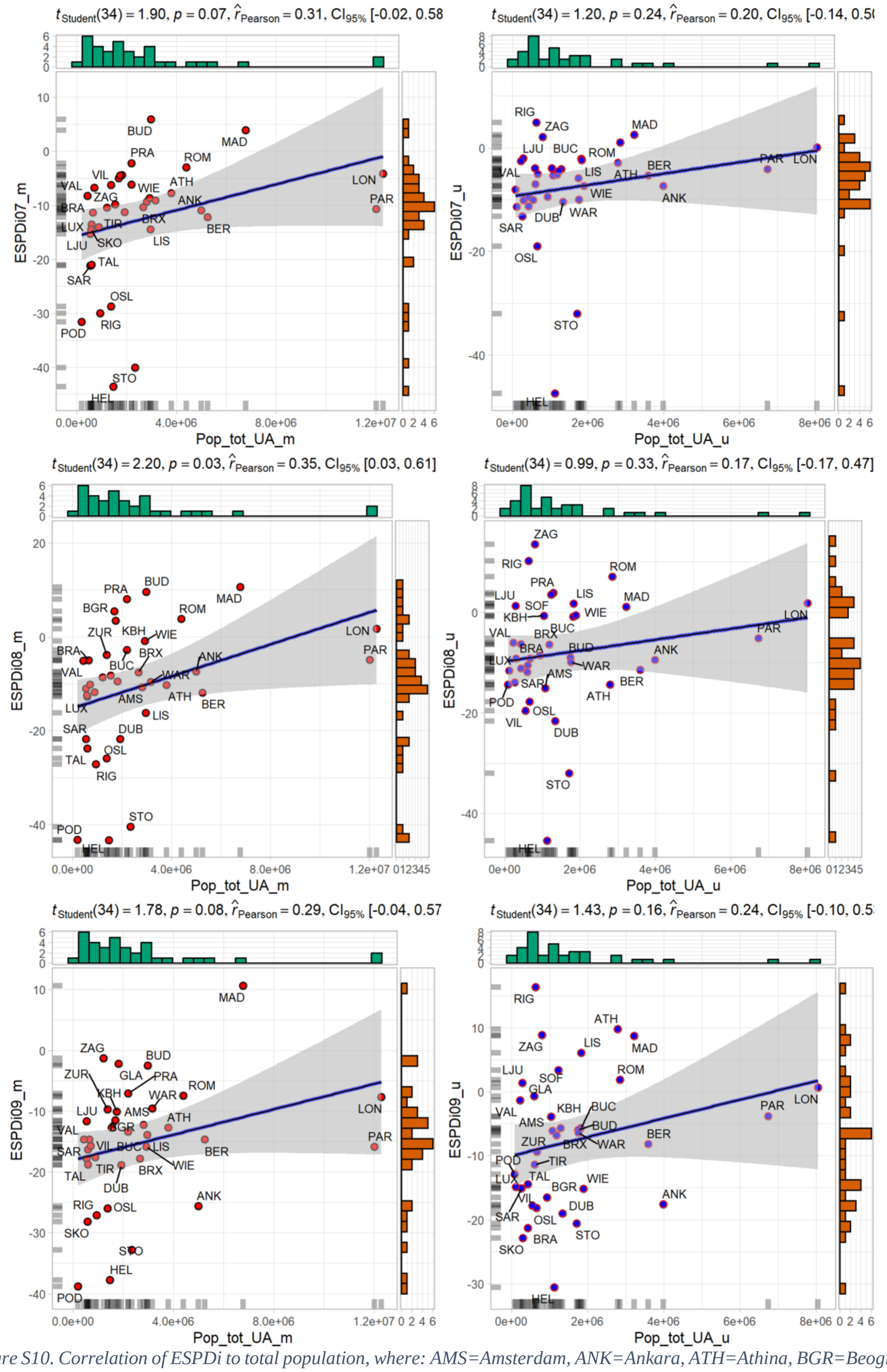

Figure S10. Correlation of ESPDi to total population, where: AMS=Amsterdam, ANK=Ankara, ATH=Athina, BGR=Beograd, BER=Berlin, BRA=Bratislava, BRX=Bruxelles, BUC=București, BUD=Budapest, DUB=Dublin, GLA=Glasgow, HEL=Helsinki, KBH=København, LIS=Lisboa, LJU=Ljubljana, LON=London, LUX=Luxembourg, MAD=Madrid, OSL=Oslo, PAR=Paris, POD=Podgorica, PRA=Praha, RIG=Rīga, ROM=Roma, SAR=Sarajevo, SKO=Skopje, SOF=Sofia, STO=Stockholm, TAL=Tallinn, TIR=Tirana, VAL=Valletta, VIL=Vilnius, WAR=Warszawa, WIE=Wien, ZAG=Zagreb, ZUR=Zürich.

Table S2. Final shortlist six ES types and their respective ESP values per each Ecosystem type (UA classes).

		Biodiversity	Drinking water	Flood protection	Air quality regulation	Water purification	Recreation and tourism	**Average ESP of each class**
**Ecosystem types [Urban Atlas 2018]**	**UA code**	**I1**	**P1**	**R1**	**R2**	**R3**	**C1**	
Continuous Urban fabric (S.L. > 80%)	11100	10	5	5	5	5	10	**7**
Discontinuous Dense Urban Fabric	11210	20	5	5	5	15	15	**11**
Discontinuous Medium Density Urban Fabric	11220	25	10	10	10	25	25	**18**
Discontinuous Low Density Urban Fabric	11230	35	10	15	15	35	35	**24**
Discontinuous very low density urban fabric	11240	40	15	20	20	45	45	**31**
Isolated Structures	11300	10	5	5	5	5	10	**7**
Industrial, commercial, public, military and private	12100	5	5	5	5	5	5	**5**
Fast transit roads and associated land	12210	5	5	5	5	5	50	**13**
Other roads and associated land	12220	20	5	10	15	10	70	**22**
Railways and associated land	12230	35	5	15	10	15	60	**23**
Port areas	12300	5	5	5	5	5	50	**13**
Airports	12400	20	5	5	5	20	10	**11**
Mineral extraction and dump sites	13100	10	5	5	5	5	10	**7**
Construction sites	13300	10	5	5	5	5	5	**6**
Land without current use	13400	30	5	30	5	30	10	**18**
Green urban areas	14100	50	5	20	30	50	60	**36**
Sports and leisure facilities	14200	30	5	5	10	30	40	**20**
Arable land (annual crops)	21000	30	5	20	20	10	40	**21**
Permanent crops	22000	30	5	20	20	10	40	**21**
Pastures	23000	50	5	30	20	40	40	**31**
Complex and mixed cultivation patterns	24000	60	30	10	30	30	60	**37**
Orchards	25000	50	5	10	30	40	50	**31**
Forests	31000	70	20	20	90	90	77	**61**
Herbaceous vegetation associations	32000	80	10	23	33	63	53	**44**
Open spaces with little or no vegetations	33000	50	10	10	10	10	40	**22**
Wetlands	40000	65	18	60	20	80	50	**49**
Water	50000	70	85	40	20	65	60	**57**
**Average ESP of each ES**		**34**	**11**	**15**	**17**	**28**	**38**	24

Table S3. Urban expansion and population growth ratios by ESPD results for each city.

Row Labels	Code	18_12_area changed	Area_tot	Urbanisation rate [m2/ha]	Pop_18_12	Pop_18_12_%	ESPD01_ALL	ESPD04_ALL	ESPD06_ALL	ESPD07_ALL	ESPD08_ALL	ESPD09_ALL
Amsterdam	AMS	4230	332721	127,1	124258	4,6	-31,4	-3,1	-6,3	-16,2	-18,4	-22,1
Ankara	ANK	15410	555809	277,3	487643	11,3	-85,8	-17,3	-13,2	-53,6	-61,7	-84,4
Athina	ATH	757	309867	24,4	-208668	-5,6	-5,8	-1,2	-1,3	-2,8	-3,7	-3,5
Beograd	BGR	1841	313079	58,8			-11,7	-9,7	-0,4	-6,0	-7,2	-9,4
Berlin	BER	4315	1748436	24,7	361221,0	7,4	-5,2	-0,4	-1,1	-3,2	-3,7	-4,2
Bratislava	BRA	2849	205160	138,9	29593,0	7,3	-31,0	-3,4	-5,5	-22,3	-20,0	-31,7
Bruxelles	BRX	1935	326569	59,2	136922,0	4,4	-15,4	-1,2	-3,8	-8,3	-9,7	-13,7
Bucureşti	BUC	3557	107810	329,9	5917,0	0,3	-56,0	-2,1	-9,8	-31,7	-27,6	-58,1
Budapest	BUD	3306	639316	51,7	64259,0	2,2	-7,2	2,3	-0,5	-3,4	-1,1	-6,5
Dublin	DUB	4332	703803	61,6	136888	7,6	-18,0	-2,3	-4,5	-10,8	-11,7	-16,7
Glasgow	GLA	3175	337685	94,0			-11,1	-0,4	-5,2	-5,7	-7,7	-4,5
Helsinki	HEL	2687	494707	54,3	108886	7,9	-21,0	-3,2	-4,8	-16,1	-19,2	-15,8
København	KBH	2751	291248	94,4	117694	6,5	-15,5	-3,6	-3,9	-7,7	-8,6	-12,6
Lisboa	LIS	2846	439327	64,8	29531	1,0	-7,1	-1,1	-0,9	-5,1	-4,7	-4,5
Ljubljana	LJU	552	233439	23,7	8911	3,2	-2,6	-0,3	-0,3	-1,7	-1,6	-1,9
London	LON	7310	698068	104,7	755176	6,5	-9,2	-1,4	0,0	-6,2	-2,9	-9,4
Luxembourg	LUX	1844	259577	71,0	76766	14,7	-17,6	-2,5	-3,7	-10,8	-11,0	-14,9
Madrid	MAD	8751	787600	111,1	89264	1,3	6,9	0,9	1,6	5,2	9,5	11,3
Oslo	OSL	2425	747375	32,4	124023	9,9	-9,6	-1,3	-2,3	-6,9	-8,2	-8,0
Paris	PAR	7355	1209772	60,8	329992	2,6	-13,4	-1,4	-2,8	-8,0	-8,3	-12,2
Podgorica	POD	812	184531	44,0			-18,9	-1,9	-3,8	-14,4	-16,9	-17,6
Praha	PRA	5772	570617	101,2	120480	5,8	-9,7	-0,8	0,7	-4,9	-1,5	-11,7
Pristina	PRI	3378	255785	132,1			-22,6	-1,2	-0,3	-10,4	-9,8	-21,2

Reykjavík	REY	1136	1297736	8,8			-2,9	-0,3	-0,4	-2,4	-2,7	-2,3
Rīga	RIG	1626	615875	26,4	-15384	-1,6	-6,4	-0,3	-1,0	-4,8	-5,1	-4,8
Roma	ROM	3480	616988	56,4	176123	4,2	-4,9	0,1	0,0	-2,4	-1,2	-4,3
Sarajevo	SAR	916	263722	34,7			-9,4	-0,7	-1,0	-6,2	-6,4	-6,8
Skopje	SKO	1523	224414	67,8			-25,2	-2,0	-4,4	-15,3	-16,5	-24,5
Sofia	SOF	2186	571690	38,2	13087	0,8	-5,0	0,4	1,3	-4,8	-2,0	-4,3
Stockholm	STO	4002	764869	52,3	216670	10,4	-17,0	-2,4	-3,5	-12,9	-15,3	-13,0
Tallinn	TAL	2661	439091	60,6	15805	4,3	-14,8	-1,7	-2,3	-10,1	-10,6	-12,0
Tirana	TIR	1434	167007	85,9			-18,7	-0,1	-2,7	-12,6	-12,6	-16,9
Valletta	VAL	207	26189	78,9	56380	14,7	-16,0	-5,4	-3,6	-10,0	-10,9	-13,3
Vilnius	VIL	3397	424662	80,0	-358	-0,1	-17,6	-1,6	-2,8	-9,6	-9,5	-18,6
Warszawa	WAR	8091	861464	93,9	118465	3,9	-18,3	-1,3	-2,6	-11,3	-11,0	-12,9
Wien	WIE	4561	918028	49,7	225774	8,2	-10,8	-1,0	-1,6	-6,2	-6,1	-10,9
Zagreb	ZAG	1599	505861	31,6	-1732	-0,1	-2,9	-0,2	-0,2	-1,5	-1,3	0,3
Zürich	ZUR	995	127634	78,0	102728	8,1	-10,7	-1,4	-4,1	-6,8	-8,0	-10,2

Table S4. Urban population growth rates of European countries based on World Bank statistics[1].

Country Name	2016	2017	2018	2019	2020	2021	21-20	20-19	19-18	18-17	17-16	5 year av.
**Albania**	58,5	59,4	60,4	61,3	62,2	63	0,86	0,89	0,91	0,94	0,97	0,92
**Austria**	58	58,1	58,3	58,6	58,8	59	0,25	0,24	0,22	0,21	0,19	0,23
**Belgium**	98	98	98,1	98,1	98,1	98,2	0,04	0,04	0,04	0,05	0,05	0,05
**Bosnia and Herzegovina**	47,6	47,9	48,3	48,7	49,1	49,5	0,41	0,4	0,39	0,37	0,36	0,39
**Bulgaria**	74,4	74,7	75,1	75,4	75,7	76,1	0,34	0,34	0,34	0,34	0,35	0,35
**Croatia**	56,5	56,7	57	57,3	57,6	57,9	0,33	0,32	0,3	0,29	0,27	0,31
**Cyprus**	66,9	66,9	66,9	66,9	66,9	66,9	0,04	0,02	-0,01	-0,03	-0,05	-0,01
**Czechia**	73,6	73,7	73,8	74	74,1	74,3	0,16	0,15	0,13	0,12	0,11	0,14
**Denmark**	87,7	87,8	87,9	88	88,2	88,3	0,13	0,13	0,13	0,12	0,12	0,13

[1] Retrieved from World Bank database (https://data.worldbank.org/indicator/SP.URB.TOTL.IN.ZS?name_desc=false)

**Estonia**	68,6	68,8	68,9	69,1	69,3	69,5	0,19	0,18	0,18	0,17	0,16	0,18
**Finland**	85,3	85,4	85,4	85,5	85,6	85,6	0,08	0,08	0,07	0,06	0,05	0,07
**France**	80	80,2	80,5	80,8	81	81,3	0,27	0,27	0,27	0,27	0,27	0,27
**Germany**	77,3	77,3	77,4	77,4	77,5	77,6	0,1	0,08	0,07	0,06	0,04	0,07
**Hungary**	70,8	71,1	71,4	71,7	72	72,3	0,31	0,3	0,3	0,29	0,29	0,3
**Iceland**	93,8	93,8	93,9	93,9	93,9	94	0,05	0,05	0,05	0,05	0,04	0,05
**Ireland**	62,8	63	63,2	63,5	63,7	64	0,26	0,25	0,24	0,23	0,22	0,24
**Italy**	69,9	70,2	70,5	70,8	71,1	71,4	0,31	0,31	0,3	0,3	0,29	0,31
**Latvia**	68,1	68,1	68,2	68,3	68,4	68,5	0,11	0,1	0,08	0,07	0,06	0,09
**Lithuania**	67,4	67,6	67,7	67,9	68,1	68,3	0,21	0,2	0,18	0,17	0,16	0,19
**Luxembourg**	90,5	90,8	91	91,3	91,5	91,7	0,22	0,24	0,25	0,26	0,27	0,25
**Montenegro**	66,2	66,5	66,9	67,2	67,5	67,9	0,34	0,34	0,34	0,34	0,34	0,34
**Netherlands**	90,7	91,1	91,5	91,9	92,3	92,6	0,34	0,36	0,39	0,42	0,45	0,4
**North Macedonia**	57,6	57,8	58	58,3	58,5	58,8	0,31	0,28	0,25	0,22	0,19	0,25
**Norway**	81,5	81,9	82,3	82,7	83	83,4	0,35	0,36	0,37	0,38	0,39	0,37
**Poland**	60,2	60,2	60,1	60,1	60,1	60,1	0,04	0,01	-0,03	-0,05	-0,08	-0,03
**Portugal**	64,1	64,7	65,3	65,8	66,4	66,9	0,54	0,55	0,56	0,56	0,57	0,56
**Romania**	53,9	54	54	54,1	54,2	54,4	0,14	0,11	0,09	0,07	0,04	0,09
**Serbia**	55,9	56	56,1	56,3	56,5	56,7	0,21	0,19	0,17	0,15	0,14	0,18
**Slovak Republic**	53,9	53,8	53,8	53,8	53,8	53,9	0,07	0,04	0,01	-0,03	-0,06	0,01
**Slovenia**	54,1	54,3	54,6	54,9	55,2	55,5	0,31	0,3	0,29	0,27	0,26	0,29
**Spain**	79,9	80,1	80,4	80,6	80,9	81,1	0,25	0,25	0,25	0,25	0,24	0,25
**Sweden**	86,9	87,2	87,5	87,8	88	88,3	0,27	0,27	0,28	0,29	0,3	0,29
**Switzerland**	73,8	73,8	73,8	73,9	74	74	0,09	0,07	0,06	0,04	0,03	0,06
**Turkiye**	74,2	74,7	75,2	75,7	76,2	76,6	0,47	0,48	0,49	0,5	0,52	0,5
**United Kingdom**	82,9	83,2	83,4	83,7	84	84,2	0,25	0,26	0,26	0,26	0,26	0,26
**Euro area**	76,6	76,8	77	77,3	77,5	77,7	0,24	0,24	0,24	0,23	0,22	0,24
**European Union**	74,2	74,4	74,6	74,8	75	75,2	0,24	0,23	0,23	0,21	0,21	0,23
**World**	54,3	54,8	55,2	55,7	56,1	56,5	0,42	0,44	0,45	0,45	0,46	0,45
